# Unveiling the Therapeutic Potential of Gallic Acid: Mechanistic Insights into the Management of Pathogenesis: A Narrative Review

**DOI:** 10.3390/ijms27031536

**Published:** 2026-02-04

**Authors:** Hajed Obaid A. Alharbi, Tarique Sarwar, Arshad Husain Rahmani

**Affiliations:** Department of Medical Laboratories, College of Applied Medical Sciences, Qassim University, Buraydah 51452, Saudi Arabia

**Keywords:** gallic acid, oxidative stress, inflammation, antidiabetic, anticancer, pathogenesis

## Abstract

Gallic acid (GA) is a natural polyphenol abundantly found in a variety of fruits, including blackberries, apples, pineapples, strawberries, bananas, and grapes. With prominent anti-inflammatory and antioxidant properties, GA effectively mitigates inflammation and oxidative stress. Furthermore, it plays a significant role in modulating various cellular processes and biological activities, ultimately inhibiting the progression of pathogenesis. This review explores the multifaceted health benefits of GA, highlighting its role as antidiabetic, anti-obesity, anti-arthritis, hepatoprotective, cardioprotective, and neuroprotective effects. Additionally, its impact on the respiratory, digestive, and reproductive systems, along with its related pathogenesis, is described. Additionally, its role as an antimicrobial is defined primarily through mechanisms such as disruption of microbial cell membranes, inhibition of efflux pumps, and antibiofilm activity. Moreover, this review provides a novel, integrative analysis of GA by unifying its mechanistic roles across various pathogenesis. It further describes the role of GA in cancer management via the modulation of signaling pathways. In addition, it demonstrates the synergistic effects of GA when used in combination with other drugs/compounds and discusses nanoformulation approaches that improve its therapeutic efficacy. However, despite significant preclinical outcomes, the clinical application of GA is limited by a shortage of human trials, low bioavailability, and an inadequate understanding of its mechanisms of action and optimal dosage. To overcome these limitations, well-designed clinical trials, in vivo studies, and advanced nanoformulation approaches are required to enhance bioavailability, elucidate mechanisms of action, and increase knowledge of safety and long-term toxicity. Addressing these gaps will enable the full exploration of GA’s benefits in disease prevention and management.

## 1. Introduction

According to the World Health Organization (WHO), almost 80% of the global population uses medicinal plants to treat or prevent diseases [1]. Natural bioactive compounds include a huge array of molecules derived from plants, marine organisms, fungi, and other natural sources [2]. Research has focused on dietary and medicinal plants due to their abundance of functional compounds, such as polyphenols, vitamins, flavonoids, and proteins, found in various parts of plants [3,4]. Polyphenols are the most common type of compound found in natural products [5]. Gallic acid (GA) is a valuable polyphenol compound, and many foods such as gallnuts, oak bark, grapes, bananas, lemons, strawberries, sumac green tea, witch hazel, pineapples, and apple peel are recognized to be abundant in GA [6]. Elevated glucose levels as well as oxidative stress directed to increased production of advanced glycation end products (AGEs), which are recognized to contribute to complications associated with diabetes. The study explored the protective effect of gallic acid (GA) against AGEs in cardiac H9C2 (2-1) cells. When cells were exposed to AGEs, there was a notable increase in reactive oxygen species (ROS), accompanied by a substantial decrease in antioxidant enzyme levels and an increase in collagen content. Moreover, cells exposed to AGEs exhibited altered mitochondrial membrane potential (MMP). However, whereas AGE-exposed cells pretreated with GA mitigated ROS release, there were no substantial changes in MMP, or collagen content. These findings propose that GA may offer protective benefits against AGEs-induced cardiovascular complications, likely due to its ability to scavenge free radicals [7].

A recent study highlights gallic acid as a promising therapeutic agent for the management of atherosclerotic cardiovascular disease (ASCVD) due to its multi-targeted mechanisms of action [8]. GA has the capability to delay the proliferation and metastasis of T24 cells while promoting apoptosis. The pro-apoptotic role is closely linked to mitochondrial dysfunction and to suppression of the PI3K/Akt/NF-κB signaling pathway [9]. Another study reports a study focused on the pharmacological properties of gallic acid (GA) and the molecular mechanisms underlying its hepatoprotective effects. Owing to its pharmacological properties, GA emerges as a promising therapeutic candidate for the prevention and management of diverse xenobiotic-induced hepatotoxicity [10].

The study evaluates the antioxidant property of the stem bark of *Elaeocarpus floribundus* Blume using an integrated approach encompassing phytochemical isolation, in vitro radical scavenging assays, ADMET-based safety profiling, as well as molecular docking analysis. The isolation and characterization of the ethyl acetate fraction of the stem bark yielded known compounds from this plant part, together with gallic acid and epigallocatechin gallate. Both compounds confirmed potent in vitro radical scavenging activity. Among them, gallic acid exhibited greater pharmacokinetic and safety profiles based on in silico ADMET predictions, with no violations of Lipinski’s rule and no predicted toxicity. Furthermore, molecular docking analyses revealed that gallic acid exhibited strong binding affinities toward superoxide dismutase (SOD) and glutathione reductase (GR), surpassing those of the corresponding reference inhibitors [11].

Despite wide-ranging research demonstrating the anticancer, neuroprotective, and cardioprotective properties of gallic acid, numerous critical knowledge gaps remain. The existing literature mainly emphasizes its role in various diseases, with limited efforts to integrate its mechanistic roles across diverse pathogenic pathways. Moreover, although numerous preclinical studies highlight its promising therapeutic potential, noteworthy inconsistencies that persist regarding optimal dosage, bioavailability, pharmacokinetics, and translational relevance to clinical applications. Therefore, this review aims to address these limitations by providing a comprehensive mechanistic and comparative analysis of gallic acid’s role in multiple pathogenesis, evaluating translational challenges, and outlining future research directions essential for clinical development. Furthermore, this review summarizes the role of gallic acid in cancer management via the modulation of key cell signaling pathways. The synergistic potential of gallic acid in combination with other drugs and bioactive compounds, as well as recent advances in nanoformulation strategies that enhance its therapeutic efficacy, are also discussed, thereby providing forward-looking insights essential for its clinical development.

## 2. Literature Search Strategy and Selection Criteria

A comprehensive literature search was carried out using various electronic databases and search engines, including Google, Google Scholar, PubMed, and Scopus, to gather scientific evidence regarding the role of gallic acid in various pathogenesis. The search encompassed publications from November 2000 to December 2025. This timeframe was chosen to include both foundational studies and the latest recent, particularly in areas such as sources, mechanisms, pharmacokinetics, synergistic effects, and nanoformulation-based therapeutic applications of gallic acid. Initially, a total of 290 records were searched. After excluding similar studies or duplicates, 235 studies were considered for screening based on their titles and abstracts, followed by a thorough evaluation of the full texts. The inclusion criteria for the studies were as follows: only peer-reviewed original research articles, review articles, and clinical studies were considered. The studies that examined the biological, pharmacological, or therapeutic effects of gallic acid were included. The articles focusing on specific activities such as antidiabetic, anti-obesity, hepatoprotective, nephroprotective, wound healing, and anticancer effects, as well as those exploring synergistic effects, pharmacokinetics, and gallic acid-based nanoformulation, were included. Only studies published in English were considered for the final review. On the other hand, case reports, editorials, letters to the editor, theses, conference abstracts, and non-peer-reviewed articles were excluded. Studies that did not directly relate to gallic acid or lacked mechanistic or therapeutic relevance were also excluded. Non-English publications were also excluded from consideration.

## 3. Structure, Sources, and Bioavailability of Gallic Acid

**Figure 1:**
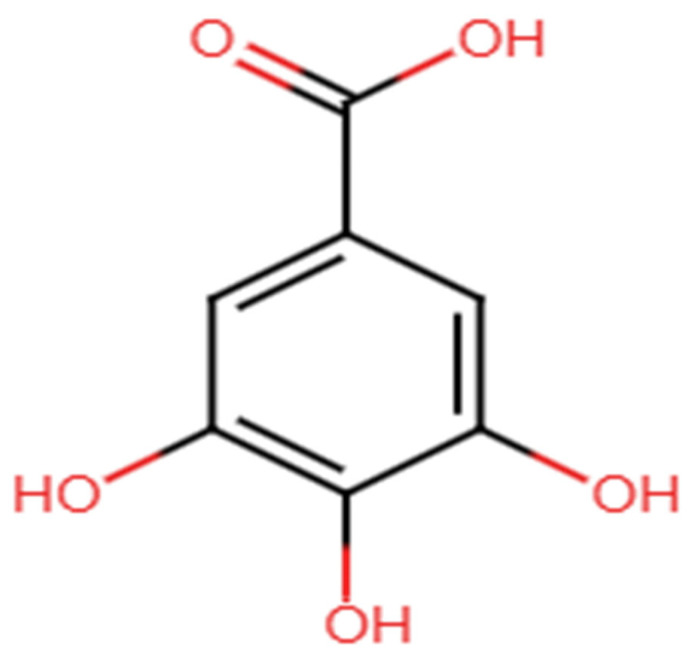
Chemical structure of gallic acid (chemical structure was made using the Chemical Sketch Tool: https://www.rcsb.org/chemical-sketch).

Gallic acid (3,4,5-trihydroxybenzoic acid) [Figure 1] is a phenolic acid with the chemical formula C7H6O5, a molecular weight of 170.12 g/mol, and is a slightly yellow crystalline powder. This compound is commonly found in the plant kingdom, often occurring in its free form or as a derivative in various food sources, including nuts, tea, grapes, and sumac [12,13,14]. Apart from plant sources, gallic acid also occurs in some beverages, for example, wine and tea [15]. Some other sources are walnuts, blackberries, apples, pineapples, strawberries, lemons, bananas, or grapes [16,17,18,19]. Carob fruit is considered one of the richest sources of gallic acid, with its content estimated to range from 23.7 mg to 164.7 mg per 100 g [20,21,22,23]. Moreover, gallic acid is also present in fruits, leaves, and bark of plants such as *Sambucus nigra* L., *Sorocea guilleminina* Gaudich, *Barringtonia racemosa* (L.) Spreng, and *Fraxinus angustifolia* Vahl, respectively [24,25,26,27].

While gallic acid demonstrates significant biological activity, its therapeutic effectiveness is limited by its low bioavailability. Findings have revealed that GA is safe and efficient, but its pharmacokinetic features, for instance, low absorption, poor bioavailability, and rapid elimination, limit its use [28,29]. A study was conducted to examine the possible differences in the pharmacokinetic processes in normal and myocardial infarcted rats after oral administration of GA monohydrate. The results exhibited that the pharmacokinetics of GA were meaningfully different between normal and pathological conditions. GA showed slower absorption into the bloodstream, and yielded 1.7-fold (50 mg/kg GA) and 1.3-fold (100 mg/kg GA) lower values of area under concentration-time curve, as well as 2.5-fold lower Cmax in myocardial infarcted rats than those in normal rats. This investigation indicates that MI has the potential to modify the PK procedure of GA [30].

The absorption of orally administered GA in rats was investigated to characterize its serum pharmacokinetic profile and intestinal absorption. Rats were administered 100 micromol/kg body weight of GA. It was reported that GA was absorbed slowly, with a t(max) for intact GA of 60 min, as well as a C (max) of 0.71 micromol/L [31]. The theaflavin skeleton is comparatively resistant to degradation by colonic bacteria with a 67% recovery being obtained after a 24 h incubation, which yielded 21 phenolic as well as aromatic catabolites. The theaflavin galloyl moiety was removed by the microbiota, and the released gallic acid (GA) further transformed to 3-O- and 4-O-methyl gallic acids, pyrogallol-1-sulfate, and pyrogallol-2-sulfate, which were excreted in urine, accounting for 94% of the intake. A number of the colonic catabolites originating from GA and theaflavins have been demonstrated to be bioactive in ex vivo as well as in vitro models with several potential modes of action [32]. Using the HPLC method, researchers investigated the pharmacokinetic differences in gallic acid (GA) between acidum gallicum tablets and black tea, both containing 0.3 mM GA, in a study involving 10 volunteers. The findings indicated that GA from both the tablets and tea was rapidly absorbed and excreted. Following the oral administration of black tea and tablets, 39.6 ± 5.1% and 36.4 ± 4.5% of the GA dose were excreted in urine as GA and 4OMGA, respectively. The relative bioavailability of GA from tea compared to that from the tablets was found to be 1.06 ± 0.26, indicating that GA is similarly available from drinking tea as it is from taking GA tablets [14]. After a single oral dose of Polygonum capitatum extract at 60 mg/kg (which corresponds to 12 mg/kg of GA), the distribution of GA was chiefly observed in the kidney tissue of rats, measuring 1218.62 ng/g. The lung tissue showed the next highest concentration of GA at 258.08 ng/g. GA levels in the liver and heart were somewhat lower than in the lungs, while the spleen showed very little GA, and none were detected in the brain tissue [33].

## 4. The Role of Gallic Acid (GA) in Different Pathogenesis and Potency of GA Across Different Disease Models

Gallic acid (GA) is a phenolic acid with the chemical formula C7H6O5. It plays a crucial role in different pathogenesis, including neurodegenerative disorders, liver, lung, digestive-system-associated pathogenesis, cardiovascular diseases, and inflammatory conditions. Preclinical studies designate that its potency can vary substantially depending on the pathogenesis types, the experimental model, and the route of administration. The gallic acid demonstrates its role in the inhibition of proliferation, cell cycle arrest, inhibition of angiogenesis and induction of apoptosis at different concentrations rang, whereas in hepatoprotective, cardioprotective, and neuroprotective studies, effective doses are different, suggesting differential sensitivity of cell types and tissues. The below text outlines the role of gallic acid in various pathogenesis, summarizing the study models, doses administered, and the effective outcomes observed. Its potency depends strongly on the disease model, dose, formulation, and combination therapy. This compilation emphasizes that gallic acid demonstrates both concentration-dependent and context-dependent effects across a range of disease models.

### 4.1. Antioxidant Potential

**Figure 2:**
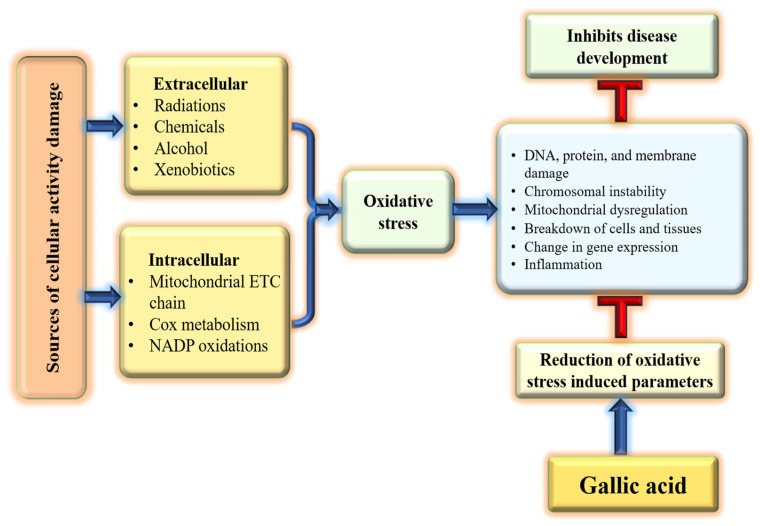
GA role in disease prevention through mitigating oxidative stress through multiple mechanisms. It includes enhancement of endogenous antioxidant enzymes, inhibition of lipid peroxidation, inflammation, prevents cellular components damage, and finally it reduces the risk of oxidative-stress-related pathogenesis.

Oxidative stress occurs when there is an imbalance between pro-oxidants and antioxidants, resulting in disrupted redox processes and damage to macromolecules [34]. The overproduction of reactive oxygen species (ROS) leads to structural changes in cellular proteins, which in turn affects their function [35,36]. Medicinal plants and their bioactive compounds show role in various disease through their antioxidant potential [37,38,39,40]. GA employs a substantial role in disease prevention through reduction of oxidative stress/scavenges free radicals and counteracts reactive oxygen species (ROS) [Figure 2].

The study investigated the effects of a metal chelator, MiADMSA, used alone or in combination with natural gallic acid, on reversing oxidative damage caused by arsenic in red blood cells. It was observed that both the MiADMSA alone and the combination with GA resulted in the reversal of oxidative and nitrosative stress indicators, a reduction in osmotic fragility, and an increase in cellular antioxidant capacity, compared to the group treated solely with arsenic [41]. The impact of prolonged administration of gallic acid (at doses of 0, 10, 20, and 40 mg/kg via gavage) was assessed in diabetic (DBT) animals through various behavioral tests, specifically the elevated plus-maze (EPM), the light-dark transition (LDT), as well as the modified forced swim test (mFST). Moreover, indirect measures of oxidative stress, including lipid peroxidation (LPO) and levels of reduced glutathione (GSH) in both the hippocampus (HIP) and prefrontal cortex (PFC) were investigated. The outcomes exhibited that DBT animals presented a reduction in the spent time in the open arms, in the end arm exploration as well as head dips when assessed in the EPM test; furthermore, a reduction in the spent time in the lit compartment of the LDT test was detected, explaining an anxiogenic-like behavior. During the mFST, an increase in the mean counts of immobility and a reduction in the mean counts of swimming as well as climbing were noticed, demonstrating depressive-like behavior. These aversive behaviors were more noticeable when compared to normoglycemic (NGL) animals and streptozotocin (STZ)-treated animals that did not become DBT. Furthermore, DBT rats exhibited an increase in the oxidative stress parameters in the PFC and HIP that was reversed by the treatment of GA (lowest dose—10 mg/kg), i.e., the treatment increased the reduced GSH and reduced the elevated LPO in the HIP and PFC. As well, GA treatment was capable to show an anxiolytic-like effect in the LDT and EPM tests [42]. The study investigated the impact of GA against restraint stress-induced oxidative damage. Findings reported that restraint stress meaningfully reduced the activities of GPX and SOD and increased MDA in the stressed rats, which were reserved by Gallic acid (100 mg/kg) treatment [43].

Farzaneh Sohrabi et al. investigated the role of GA in the inflammatory process and the probable signaling pathway in the elastase-induced emphysema. Oxidative stress indexes such as antioxidant enzyme activity and malondialdehyde were determined. The gene expression levels of nuclear factor (erythroid-derived 2)-like 2 (Nrf2), nuclear factor kappa-light-chain-enhancer of activated B cells (NF-κB), and heme oxygenase-1 (HO-1), were measured as key controllers of antioxidant as well as inflammation systems. The porcine pancreatic elastase (PPE) group displayed pulmonary edema and noteworthy alterations in arterial blood gas values, which were associated to reduced antioxidant enzyme activity and changes in the expression of the Heme Oxygenase-1 (HO-1), Nuclear factor erythroid 2-related factor 2 (Nrf2), and nuclear factor kappa-B (NF-κB) in comparison to the control group. Co-treatment with gallic acid almost restored all of these changes to normal levels. The outcomes established that elastase-induced emphysema leads to injuries of the lung, which are linked with inflammation and oxidative stress. Furthermore, the outcomes proposed that GA as a natural antioxidant agent can modulate the Nrf2 signaling pathway to defend the lung against elastase-caused emphysema [44]. The ameliorative potential effect of gallic acid against paraquat-induced renal injury and oxidative stress was examined. Findings of this work exhibited treatment with GA after exposure to paraquat led to a noteworthy elevation in renal catalase, vitamin C, and superoxide dismutase levels plus a remarkable decrease in the MDA as compared to paraquat-only-treated rats [45]. GA treatment (50, 100, and 200 mg/kg) raises the antioxidant defense against BCCA occlusion-caused ischemia/reperfusion in rats, demonstrating that it holds neuroprotective potential [46]. The administration of cyclophosphamide led to an increase in cardiac and renal malondialdehyde levels, as well as hydrogen peroxide production. Likewise, the activities of catalase, glutathione-S-transferase, glutathione peroxidase, and levels of reduced glutathione decreased following cyclophosphamide treatment. Additionally, an increase in serum myeloperoxidase activity was observed in rats treated solely with cyclophosphamide. Treatment with gallic acid restored both enzymatic and non-enzymatic antioxidants and reduced the cardiotoxic and nephrotoxic effects of cyclophosphamide through its free radical scavenging activity [47]. Animals treated with lindane showed increased levels of lipid peroxidation, along with a corresponding decrease in the levels of non-enzymatic antioxidants as well as enzymatic antioxidants in heart tissue. The results suggest that gallic acid and quercetin offer protection against lindane-induced myocardial damage, likely by preserving the levels of endogenous antioxidant enzymes [48]. The protective effect of GA against the impact of advanced glycation end products (AGEs) in cardiac H9C2 (2-1) cells was assessed. When the cells were exposed to AGEs, there was a release of reactive oxygen species (ROS) and a decrease in antioxidant enzyme levels. However, in cells pretreated with gallic acid, the release of ROS was prevented. There is evidence that gallic acid plays a protective role against cardiovascular complications [7]. The protective effects of GA against CCl4-induced liver fibrosis were examined. GA upregulated the antioxidant genes expression, specifically catalase and superoxide dismutase. Therefore, the use of GA as a natural antioxidant holds promise for improving liver diseases [49].

### 4.2. Anti-Inflammatory Potential

Inflammation is a crucial factor in the development of various chronic illnesses, such as neurodegenerative diseases, gastrointestinal disorders, diabetes, cardiovascular diseases, arthritis, and cancer [50]. The effects of natural compounds in different pathogenesis have been documented through various mechanisms [51,52,53,54,55]. The role of gallic acid in pathogenesis has been reported through different mechanisms [56]. The impact of GA on airway inflammation and the expression of Th1, Th2, and Th17 cytokines were examined in a mouse model of allergic rhinitis (AR) induced by ovalbumin (OVA). GA was found to reduce nasal allergic symptoms, decrease goblet cell hyperplasia and eosinophil infiltration in the nasal mucosa, and lessen the thickness of the nasal mucosa. Additionally, GA lowered the levels of interleukins (IL)-4, IL-5, IL-13, and IL-17 in nasal lavage fluid (NALF) and decreased the concentrations of OVA-specific IgE, IgG1, and IgG2a in serum. Conversely, GA increased the expression of interferon-gamma and IL-12 in NALF [57]. Ovalbumin led to an elevated infiltration of pro-inflammatory cells, whereas gallic acid lessened this infiltration and improved airway hyperresponsiveness. Additionally, gallic acid lowered the levels of IL-5, 13 in bronchoalveolar lavage fluid and reduced the expression of IL-33 in the lungs [58]. The study assessed the preventive effects of GA against liver fibrosis induced by carbon tetrachloride (CCl4). It was reported that GA significantly reduced serum hepatic enzyme levels, diminished the expression of pro-inflammatory cytokines such as tumor necrosis factor-alpha (TNF-α), interleukin 1 beta (IL-1β), cyclooxygenase 2 (COX-2), and interleukin 6 (IL-6) [49].

The study determined the effects of GA on intestinal mucositis induced by methotrexate (MTX) in rats. Findings revealed that GA treatment meaningfully decreased the MTX-induced increase in serum levels of MDA, IL-2, and IL-6. Additionally, histopathological findings indicated that MTX caused damage to intestinal tissues, whereas gallic acid remarkably improved these pathological changes. Overall, the results propose that gallic acid can alleviate oxidative stress and inflammatory markers, as well as provide protection against histopathological damage in the small intestine of rats subjected to MTX [59]. The effects of GA were examined in a neuroinflammatory rat model, revealing that treatment with gallic acid (100 mg/kg) meaningfully reduced the LPS-induced elevations of interleukin-1β and iNOS in the LPS-infused substantia nigra of the rat brain, compared to the vehicle-treated rats [60]. The potential anti-inflammatory role of gallic acid was tested. The anti-inflammatory factors IL-4 and IL-10 levels were decreased, and pro-inflammatory factors were increased in the model group as compared to the control group. However, there was a substantial decrease in pro-inflammatory in the gallic acid treatment group. In addition, the anti-inflammatory cytokines were evidently induced when treated with GA [61]. A study based on rheumatoid arthritis reported that treatment of GA modulated the dysregulation of inflammation-related cytokines and reduced the overexpression of matrix metalloproteinases (MMPs) [62]. The study was made to investigate the gastroprotective effect GA on ethanol-induced gastric ulcer in rats. Gastric ulcers were induced by absolute ethanol in rats. Pretreatment with GA showed a notable reduction in inflammatory cytokines markers, ulcer index, and TBARS compared with ethanol treated groups [63].

### 4.3. Hepatoprotective Effects

**Figure 3:**
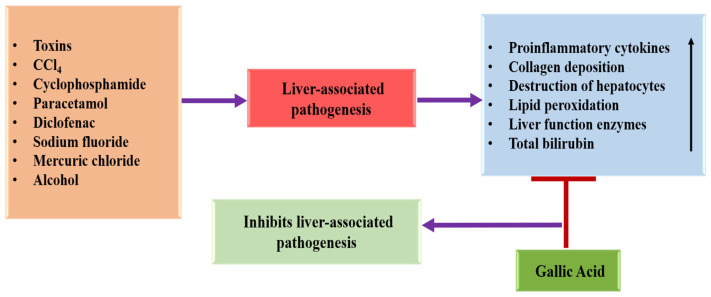
Hepatoprotective role of GA through the reduction of oxidative stress. GA (gallic acid) exerts hepatoprotective activity by reducing oxidative stress, and inhibiting lipid peroxidation. These effects collectively preserve hepatocellular integrity, prevent oxidative damage, and inhibit liver associated pathogenesis. An upward arrow (↑) denotes increased.

The liver is a decisive organ accountable for vital physiological functions with metabolism, synthesis, as well as detoxification [64]. Numerous substances, including drugs and alcohol, target hepatocytes, leading to the release of various mediators such as inflammatory factors and cytokines. This process triggers necrosis, apoptosis, and fibrosis in the liver [65,66], ultimately resulting in liver damage. Plant derivatives or bioactive compounds have a well-established role in protecting the liver by reducing liver function enzymes, as well as preserving the structure of hepatocytes [67,68,69]. Hepatoprotective potential of gallic acid through different mechanisms is described in Table 1 and Figure 3. The liver protective potential of gallic acid against liver inflammation was studied. Results revealed that decreased SOD levels were associated with increased malondialdehyde and ATPases (Ca^2+^/Mg^2+^) in N′-Nitrosodiethylamine (NDEA)-treated rats. It showed activated HSCs, deposition of collagen, and periportal and bridging fibrosis in NDEA-treated liver samples. Gallic acid supplements restore the liver functioning in rats damaged with NDEA, possibly by inducing Nrf2-mediated antioxidant enzymes and decreasing the inflammatory mediators COX-2 via the NF-κB inhibition pathway [70]. Another study based on carbon tetrachloride (CCl4) induced liver fibrosis designed to evaluate the hepatoprotective role of gallic acid. It was reported that gallic acid decreases serum hepatic enzymes, down-regulates the expression of pro-inflammatory cytokines, IL-1B, IL-6, tumor necrosis factor-alpha (TNF-α), and cyclooxygenase 2 (COX-2) [49]. Oxidative stress-induced hepatic injury caused by cyclophosphamide (CP) was evaluated by measuring reduced glutathione (GSH) levels and superoxide dismutase (SOD) activity, as well as by examining histological changes in hepatic tissue of mice. It was found that pretreatment with gallic acid significantly reduced DNA strand breaks and the frequency of micronuclei (MN) induced by CP and offered protection to the hepatic tissue [71]. The results indicated that treatment with gallic acid significantly reduced the levels of alkaline phosphatase, aspartate aminotransferase, alanine aminotransferase, and lipid peroxidation in liver tissues, which were increased by mercuric chloride. The liver of rats exposed to mercuric chloride showed cell degeneration with mild cytoplasmic blebbing, vacuolation, binucleated cells, and notable dilation of the sinusoids. It can be concluded that GA aids in restoring the activity of antioxidant enzymes as well as tissue markers in these mercuric chloride-treated rats, probably by neutralizing free radicals [72]. A study was conducted to evaluate the hepatoprotective potential of gallic acid. It was reported that mice exposed to paracetamol exhibited elevated levels of TNF-α, increased lipid peroxidation, and heightened activities of liver marker enzymes. However, treatment with gallic acid reversed these changes due to its antioxidant properties [73]. The findings indicated that pretreatment with gallic acid restored the changes in oxidative stress and serum parameters caused by sodium fluoride in hepatic tissue. Additionally, treatment with gallic acid meaningfully reduced the levels of TBARS [74]. In CCl4-treated groups (both acute and chronic), a meaningful increase in serum hepatic enzyme activities, as well as a disruption in antioxidative status, and histopathologic alterations, while the treatment with gallic aid improved histopathologic alterations, restored serum liver enzyme activities, decreased lipid peroxidation, and increased glutathione (GSH) levels. In the acute model, gallic acid and dodecyl gallate enhanced the activities of liver antioxidant enzymes. Following treatment with gallic acid and dodecyl gallate, p53 gene expression increased approximately 3.5-fold, which may lead to the elimination of damaged hepatocytes and help prevent lifelong liver failure [75].

The hepatoprotective effects of gallic acid were assessed in a rat model of diclofenac-induced liver toxicity. The results indicated that treatment with gallic acid led to an elevation in SOD, CAT, GSH, and GPx, and a decrease in protein carbonyl, total bilirubin, MDA, aspartate aminotransferase (AST), alkaline phosphatase (ALP), and alanine transaminase (ALT), compared with the diclofenac-treated group [76]. Hepatotoxicity was indicated by elevated levels of malondialdehyde (MDA) and reduced antioxidant enzyme activities in the livers of rats treated with sodium fluoride. However, when gallic acid was co-administered, improvements in these parameters were observed. This study clearly demonstrated that gallic acid has a protective role against oxidative damage in rats intoxicated with sodium fluoride [77]. The study was designed to inspect the protective effects of GA and caffeine against hepatotoxicity induced by CCl4. Treatment with CCl4 led to a substantial increase in ALT, AST, ALP, and LDH, as well as elevated hepatic lipid peroxidation products measured by MDA. This was accompanied by a notable decrease in hepatic catalase (CAT) activity and serum total antioxidant capacity. GA, as well as caffeine, were assessed for their hepatoprotective and antioxidant properties based on their impact on total antioxidant capacity, serum liver function enzymes, hepatic antioxidant activities, and mtDNA content, which approached normal levels in rats treated with CCl4. The findings designate that both GA and caffeine exhibit strong hepatoprotective effects against CCl4-induced hepatotoxicity and are capable of protecting mtDNA from depletion [78].

### 4.4. Neuroprotective Activity

**Figure 4:**
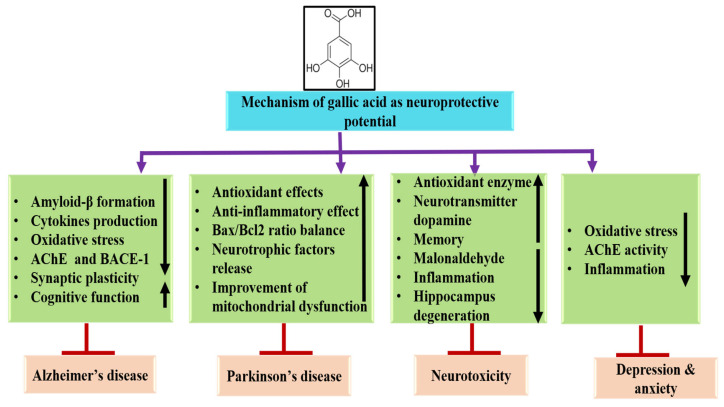
Neuroprotective role of GA through different mechanisms. It exerts neuroprotective effects by reducing oxidative stress within neural tissues, and improvement of memory. By preserving neuronal integrity, GA inhibits neuro-associated pathogenesis. An upward arrow (↑) denotes increased, and a downward arrow (↓) denotes decreased.

Gallic acid serves a vital role as a neuroprotective agent through various mechanisms. This natural compound helps to protect neural cells from oxidative stress by scavenging free radicals. Neuroprotective potential of gallic acid through different mechanisms is described in Table 2 and Figure 4. A study revealed that the two-vessel occlusion (2VO) significantly impaired spatial memory performance, in the Morris water maze. The findings indicated decreases in both non-enzymatic and enzymatic antioxidant levels, along with an increase in malondialdehyde (MDA) levels in the hippocampus and frontal cortex of the vehicle-treated group. Additionally, chronic administration of gallic acid restored the total thiol and GPx contents and spatial memory and also reduced MDA levels in these tissues. The results establish that gallic acid has beneficial effects against 2VO-induced cognitive deficits [79]. It was reported that rotenone-induced muscle weakness, in addition to motor coordination deficit and an increase in neuronal degeneration were presented in both the striatum and substantia nigra parvocellularis (SNc). Moreover, the deteriorating effects of rotenone were improved by gallic acid treatment, and gallic acid displayed an inhibitory effect on the lipid peroxidation increment [80]. It was reported that intracerebroventricular-streptozotocin (ICV-STZ) injection reduced the passive avoidance and spatial memory performance linked with decreased non-enzymatic and enzymatic activities and increased the level of thio-barbituric acid reactive species in the hippocampus and cerebral cortex. The chronic administration of gallic acid substantially inhibited cognitive deficits and biochemical alterations in the ICV-STZ rats [81]. The findings indicated that fresh green tea leaf (GTL) and GA efficiently reduced the severity of seizure classes and lipid peroxidation in male FVB mice experiencing status epilepticus (SE). Both GTL extract and GA showed a dose-dependent protective effect against KA-induced stress in PC12 cells. In exploring the protective mechanisms, it was observed that GTL and GA led to reductions in Ca^2+^ release, reactive oxygen species (ROS), and lipid peroxidation in KA-stressed PC12 cells. Moreover, the expression levels of MAPKs, COX-2, and RhoA were elevated in PC12 cells subjected to KA stress; however, GTL and GA meaningfully lowered the levels of COX-2 and p38 MAPK, while RhoA levels remained unchanged. Overall, these results suggest that GTL and GA offer neuroprotective benefits against excitotoxicity and could have potential therapeutic applications in epilepsy [82]. Oral administration of gallic acid significantly improved spatial reference memory and spatial working memory in 4-month-old APP/PS1 mice. Additionally, it notably reduced the more severe deficits observed in 9-month-old APP/PS1 mice regarding reference memory, spatial learning, short-term recognition, and spatial working memory [83]. GA has the potential to serve as a practical option for mitigating neuronal loss and neurobehavioral disorders in the hippocampus and the prefrontal cortex caused by restraint stress, mainly by reducing oxidative damage [43]. The study investigated the protective effects of gallic acid (GA) against neurotoxicity. Gallic acid significantly lowered the elevated levels of malondialdehyde (MDA) and restored the activity of glutathione peroxidase (GPx) and glutathione (GSH) in various regions of the brain. By enhancing endogenous antioxidant activity, gallic acid contributed to the recovery from sodium arsenite-induced neural and behavioral dysfunction [84].

It was observed that gallic acid treatment effectively protected against cell death induced by oxygen-glucose deprivation and/or reoxygenation (OGD/R). Furthermore, gallic acid treatment diminished neuroinflammation and neuronal loss while enhancing motor and cognitive functions [85]. The administration of GA meaningfully reduced the reduction in calcium transients in ACC neurons. GA also confirmed its ability to alleviate oxidative stress in the brain and enhance cognitive function impaired by sleep deprivation [86]. A recent study showed that cadmium chloride (CdCl_2_) causes brain impairments in experimental animals. Gallic acid was found to counteract several effects induced by CdCl_2_, including the inhibition of butyrylcholinesterase and acetylcholinesterase, elevated levels of neurotransmitters, diminished glutathione levels, decreased Na^+^/K^+^ ATPase activity, and increased malondialdehyde (MDA) levels. Additionally, gallic acid helped lower neuroinflammatory markers such as interleukin-6 and nitric oxide [87].

### 4.5. Cardioprotective Potential

**Figure 5:**
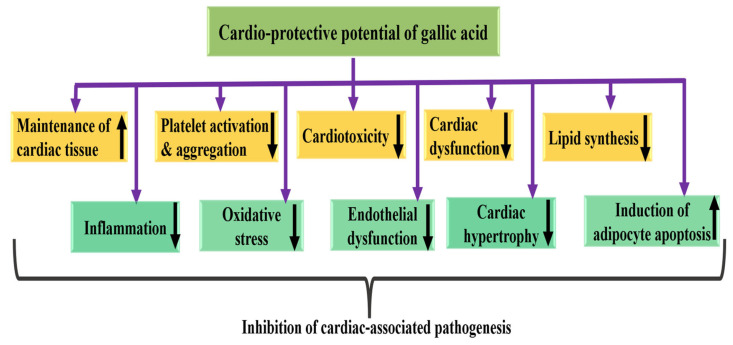
Cardioprotective role of GA through different mechanisms. It exerts cardioprotective effects tissues, and improvement of cardiac function. By preserving cardio integrity, GA inhibits cardio-associated pathogenesis. An upward arrow (↑) denotes increased, and a downward arrow (↓) denotes decreased.

The cardioprotective potential of gallic acid is attributed to numerous mechanisms that help protect heart cells from damage. The cardioprotective potential of gallic acid through various mechanisms is described in Table 3 and Figure 5. The effect of gallic acid on cardiac marker enzymes, troponin-T, lipid peroxidation products, and LDH isoenzyme patterns in isoproterenol (ISO)-induced myocardial infarction was studied. Increased levels of marker enzymes and troponin-T in the serum indicated myocardial damage caused by ISO. Furthermore, lipid peroxidation products were elevated in both plasma and heart tissue, while the activities of enzymatic antioxidants in the heart and non-enzymatic antioxidants in both plasma and heart were reduced in ISO-induced animals. Gallic acid pretreatment demonstrated a substantial protective effect on all analyzed biochemical parameters [88]. The treatment with doxorubicin increased the levels of serum cardiac as well as lipid biomarkers, which were reduced by the treatment of gallic acid. Moreover, the histopathological study revealed that gallic acid restored the myocardial cells to normal [89]. Another study showed that gallic acid improved cardiac function in the post-ischemia/reperfusion period in isoproterenol-induced hypertrophic hearts. Additionally, it reduced levels of LDH and CK-MB and alleviated cardiac hypertrophy in rats injected with isoproterenol. The administration of gallic acid also boosted the expression of the SERCA2a gene and elevated SOD levels in the hypertrophic hearts [90]. It was reported that gallic acid dose-dependently reduced cardiomyocyte size and ANP gene expression after the Ang II stimulation. Moreover, gallic acid activates autophagy in cardiomyocytes. Administration of gallic acid in mice decidedly improves transverse aortic constriction (TAC)-induced cardiac dysfunction and reduces pathological changes [91]. The role of gallic acid (GA) against doxorubicin-induced cardiotoxicity was examined. TNF-α and Cox-2 expression reduced, pathologic tissue damage reduced, and MDA level reduced in the gallic acid and doxorubicin group compared to the doxorubicin group. This finding advocate that GA has a protective effect against doxorubicin-caused cardiotoxicity [92]. Another study reported that levels of the xanthine oxidase (XO) enzyme were significantly elevated in heart homogenates of rats administered isoproterenol (ISO) compared with normal rats. However, pretreatment with maslinic acid (MA) and GA in ISO-treated rats effectively reduced XO enzyme levels to near normal levels, suggesting a protective effect of MA and GA against myocardial necrosis. These in vivo findings were further corroborated by an in silico molecular docking study, which demonstrated the inhibition of the XO enzyme through the formation of an enzyme-ligand complex with GA and MA [93].

Research indicated that gallic acid modulated the lipid profile and reduced LDL levels compared with cadmium-treated rats. Furthermore, gallic acid effectively inhibited the activity of cardiac marker enzymes, including CK-NAC and CK-MB. Furthermore, gallic acid attenuated lipid peroxidation and attenuated lipid peroxidation. Fibrotic proliferation and ventricular hypertrophy induced by cadmium were decreased by gallic acid. Additionally, gallic acid significantly lowered the expression of the profibrotic factor TGF-β. It also decreased the pro-inflammatory gene MCP-1 in rats intoxicated with cadmium [94]. The activity of lactate dehydrogenase and creatine kinase-MB increased in isoproterenol (ISO)-induced cardiotoxic rats. The levels of lipid peroxidation products increased, and the level of reduced glutathione was decreased in the plasma and heart activities of lysosomal enzymes increased in isoproterenol-induced cardiotoxic rats. Moreover, pretreatment with gallic acid in ISO-treated rats prevented changes in the levels of lipid peroxidation products, activities of cardiac marker enzymes, activities of lysosomal enzymes, and reduced glutathione [95]. The potential role of gallic acid and coadministration with GSK650394 on ischemic complications in a cardiac ischemia/reperfusion (I/R) injury animal model was investigated. The results showed that both drugs boosted the activity of endogenous antioxidant enzymes and boosted total antioxidant capacity more effectively than either drug alone. Additionally, markers for heart damage, including MDA levels, infarct size, reactive oxygen species (ROS), and SGK1 gene expression, were significantly reduced compared to the ischemic group [96]. Exposure of rats to doxorubicin resulted in a significant decrease in the cardiac antioxidant defense system, along with elevated levels of CK-MB and LDH. Furthermore, pretreatment with gallic acid alleviated the ECG abnormalities associated with doxorubicin and prevented cardiac damage [97].

### 4.6. Renoprotective Effects

Renal disease is a serious issue that demands urgent consideration, as its prevalence is rising worldwide. Current treatments are expensive and have adverse effects, highlighting the need for effective and more affordable alternatives to manage or treat this condition. Numerous functional and bioactive compounds derived from natural products have been recognized for their significant properties in treating chronic kidney disease [98]. The renoprotective potential of gallic acid through various mechanisms is described in Table 4. The protective effects of gallic acid on crystal-induced renal injury were checked. The administration of gallic acid reduces the adhesion of calcium oxalate (CaOx) stones and prevents renal deposition. Additionally, gallic acid improves renal tubular injury [99]. The ameliorative effect of gallic acid against paraquat (PRQ)-induced renal injury was measured. It was reported that treatment with gallic acid after exposure to PRQ caused an elevation in renal vitamin C and a reduction in the serum protein carbonyl, creatinine, MDA, and uric acid in comparison with the PRQ-only-treated rats. The histological changes improved with the administration of gallic acid [45]. The role of gallic acid in mitigating renal injury caused by lipopolysaccharide in rats was examined. Compared with the model group, various doses of gallic acid can improve the expression of renal biochemical markers. Additionally, gallic acid reduces kidney injury and activates AMPK/SIRT1 [100]. The effects of GA on sodium arsenite (SA)-induced renal as well as hepatic toxicity were examined. It was reported that treatment with GA meaningfully improved the changes in histopathological and hematological parameters caused by SA. These protective effects were related to a reduction in the elevated levels of MDA, NO, and IL-1β induced by SA, alongside a decrease in GSH levels and the activity of CAT, GPx, and SOD. These results suggest that GA may mitigate SA-induced toxicity in the kidneys and liver by scavenging reactive oxygen species [101].

The co-treatment of AMCs plus gallic acid in acute kidney injury (AKI) rats decreased BUN and creatinine and ameliorated kidney injury parameters, improved oxidative stress markers, and increased antioxidant enzymes in the gallic acid (GA) and AMCs group [102]. The role of gallic acid in mitigating kidney fibrosis, oxidative stress, and inflammation in a glucolipotoxicity-induced diabetic model was investigated. Gallic acid treatment increases the activity of antioxidant enzymes, while reducing lipid accumulation in the kidneys, demonstrating a protective effect against high-fat diet (HFD)-induced steatosis. GA improved nephropathy by restoring renal function and protecting against hyperinsulinemia, glomerular fibrosis, and basement membrane thickening in mice fed a high-fat diet [103]. Experiments were made to examine the role of gallic acid in diclofenac (DIC)-induced renal injury. It was demonstrated that treatment with gallic acid caused important improvements in abnormalities of DIC-induced serum biochemical parameters and oxidative stress. GA oral administration ameliorated histological changes [104]. Another study was performed to examine the effects of methotrexate (MTX) on the kidneys and to evaluate the role of gallic acid (GA). It was revealed that in the MTX and gallic acid group, SAA and CRP showed no expression, and only a limited number of PGE-2 and TNF-α positive tubular epithelial cells were observed, indicating anti-inflammatory effects [105]. It was reported that cisplatin-induced nephrotoxicity was evident, as indicated by elevated levels of urea, creatinine, uric acid, and malondialdehyde (MDA) in renal tissue. Administration of gallic acid helped to modulate the markers of nephrotoxicity, gene expression changes, and histopathological damage [106]. The role of gallic acid (GA) against cisplatin-induced nephrotoxicity was examined. It was reported that GA treatment resulted in a decrease in kidney malondialdehyde (MDA) content and an increase in total antioxidant capacity (TAC). GA also lowered the levels of inflammatory factors and alleviated kidney dysfunction, as evidenced by a reduction in creatinine levels [107]. A study reported that methotrexate (MTX) caused an increase in urea and creatinine, as compared with the control. Pretreatment and co-treatment with gallic acid effectively mitigated the biochemical changes induced by MTX [108]. GA showed protective effects against HgCl_2_-induced renal damage, likely through scavenging free radicals, and reducing oxidative stress [109].

**Table 4:** Renoprotective potential of gallic acid based on an animal model through different mechanisms. This table summarizes the effects of GA as renoprotective.

**Renoprotective activity**	**Study Model**	**Doses**	**Outcomes**	**Refs.**
	Paraquat-induced renal injury model	50, 100 mg/kg	° Kidney degeneration and lymphocytic cell infiltration reduced by GA treatment	[45]
	Lipopolysaccharide-induced renal injury model	50, 100 mg/kg	° GA improved the pathological changes of renal tissue ° GA can improve renal function ° GA alleviated inflammation	[100]
	Sodium-arsenite-induced hepatic toxicity model	10, 30 mg/kg	° Administration of GA inhibited elevation of BUN and Cr and increased antioxidant enzyme activity ° GA reduced glomerulus diameter and elevation in proximal tubule damage	[101]
	Glucolipotoxicity-induced diabetic model	100 mg/kg	° GA improved glomerular damage and renal function ° GA treatment improved pathological changes and ameliorated lipid peroxidation	[103]
	Diclofenac-induced renal injury model	50, 100 mg/kg	° GA reduced oxidative stress and improved histopathological changes	[104]
	Methotrexate-induced kidney damage model	100 mg/kg	° Gallic acid ameliorated MTX induced pathologies	[105]
	Cisplatin-induced Nephrotoxicity model	50 mg/kg	° Administration of GA modulated the markers of nephrotoxicity, gene expression changes ° GA improved histopathological damage	[106]
	Cisplatin-induced nephrotoxicity model	20, 40 mg/kg	° GA also reduced the inflammatory factor levels ° It improved kidney dysfunction	[107]
	Methotrexate-induced and nephrotoxicity model	20 mg/kg	° GA protected against MTX-induced nephrotoxicity	[108]
	Mercuric-chloride-induced kidney injury model	50, 200 mg/kg	° GA had a protective effect against renal damage	[109]

### 4.7. Antidiabetic Potential

Diabetes is a metabolic condition that impacts a significant number of people globally. While existing treatment methods are effective, they can also lead to adverse side effects. In this context, natural compounds and their bioactive compounds have demonstrated their effectiveness in managing diabetes through various mechanisms [54,110,111]. The antidiabetic potential of gallic acid through various mechanisms is described in Table 5. The potential role of GA in diabetic nephropathy induced by Methylglyoxal (MG) in mice was inspected. MG increased miR-192 and miR-204 expression, albuminuria, malondialdehyde, and Nrf2 in diabetic groups, while GA showed a role in the decrease of all such parameters. Furthermore, in diabetic groups, there was a reduction in antioxidant enzymes, glyoxalase 1, and miR-29a expression, whereas treatment with gallic acid led to an increase in these levels. The study concluded that GA mitigated diabetic nephropathy induced by methylglyoxal by improving biochemical parameters, addressing histopathological changes, and reducing oxidative stress [112]. Gallic acid (GA) promotes liver fat production by downregulating miR-34a-5p via the inhibition of NFE2L2 expression. This indicates that GA or an agent that activates NFE2L2 could have a potential therapeutic role in diabetic fatty liver disease [113]. Antidiabetic potential of gallic acid has been confirmed by previous studies [114,115,116]. In streptozotocin-induced diabetic rats fed a high-fat diet, administration of gallic acid reduced fasting blood glucose, body weight gain, and plasma insulin levels. It also brought various biochemical parameters of the diabetic-treated rats back to near-normal levels while exhibiting cytoprotective effects on pancreatic β-cells. Gallic acid meaningfully increased the expression levels of PPARγ in the adipose tissue of treated rats compared to untreated diabetic rats [117]. Study findings reported that the combination of gallic acid (GA) and metformin (MET) showed a more potent renal protective outcome in diabetic mice than GA-alone or MET-only [118]. The oral administration of gallic acid showed substantial protective effects on all examined biochemical parameters. Histopathological analysis of the pancreas established the protective effects of GA in diabetic rats [119].

### 4.8. Anticancer Potential

**Figure 6:**
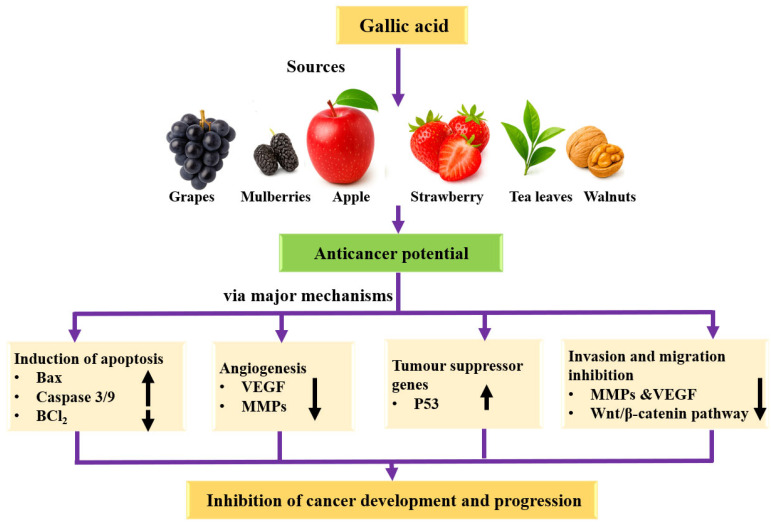
Anticancer activity of GA through various mechanisms of action. GA exhibits anticancer properties by reducing angiogenesis and induction of apoptosis and finally contributes to suppression of tumor progression. An upward arrow (↑) denotes upregulation, and a downward arrow (↓) denotes downregulation.

Cancer is a group of malignant disorders that develop in various tissues and organs, characterized by uncontrolled growth and the ability to spread to distant sites [120,121]. Cancer has become one of the main causes of human morbidity and mortality in the world [122]. The current mode of treatment, based on surgery, chemotherapy, and radiotherapy, is effective but also causes adverse effects. In vivo and in vitro studies confirmed that natural compounds or their compounds inhibit cancer through different mechanisms. In this regard, gallic acid has proven its role in cancer management through different mechanisms [Table 6 and Figure 6].

Bladder cancer-based findings demonstrated that inhibition of GA on T24 cells viability was meaningfully higher than 5-FU with the same concentration as well as time, particularly for stimulation for 24 and 48 h. The half inhibitory concentration (IC50) values for GA stimulating T24 cells over 24, 48, and 72 h were 21.73, 18.62, and 11.59 µg/mL, respectively. In comparison, the IC50 values for 5-FU were 102.04, 35.63, and 20.01 µg/mL. When compared to the control group, the viability of T24 cells in the GA-treated group was meaningfully reduced, indicating that GA effectively inhibited T24 cell viability in a time- and concentration-dependent manner. As GA stimulated T24 cells for 24 h, the inhibition rate of GA with 6.25 and 25 µg/mL was 6.89 and 65.77%, correspondingly. These outcomes indicated that GA meaningfully inhibited the viability of T24 cells. Aside from this, it meaningfully inhibited the proliferation of T24 cells and effectively blocked their cell cycle at the S phase. GA triggered apoptosis in T24 cells, which was related to an accumulation of ROS and depolarization of the mitochondrial membrane potential (MMP). The treatment with GA led to a marked increase in the expression levels of Cleaved caspase-3, Bax, P53, and Cyt-c proteins, while it decreased the levels of P-PI3K, Bcl-2, P-Akt, P-IκBα, P-IKKα, and P-NF-κB p65 proteins in T24 cells. Additionally, GA significantly enhanced the expression of Caspase-3, Bax, P53, and Cyt-c genes, while downregulating the expression of Bcl-2, Akt, PI3K, and NF-κB p65 genes. Moreover, GA considerably impaired the migration and invasion capabilities of T24 cells by inhibiting VEGF protein expression [9]. The study was made to examine the signaling pathways related with GA induced matrix metalloproteinase-2 (MMP-2)/MMP-9 downregulation in human leukemia K562 cells. GA induced β-TrCP upregulation evoked Bcr/Abl degradation, whereas overexpression of Bcr/Abl attenuated gallic acid induced MMP-2/MMP-9 downregulation. GA treatment repressed JNK1-mediated ATF-2 phosphorylation and Akt/ERK-mediated c-Fos phosphorylation. c-Jun inactivation was mediated via GA-induced Akt/ERK and JNK inactivation. The data designate that MMP-2 as well as MMP-9 downregulation in GA-treated K562 cells are mediated via suppression of JNK1-mediated c-Jun/ATF-2 as well as Akt/ERK-mediated c-Jun/c-Fos pathways, correspondingly [123]. A recent study focusing on oral cancer has found that GA meaningfully hinders the migration and invasion of SCC-4 cells, as demonstrated by the results from both the wound healing assay and the Matrigel Cell Migration Assay and Invasion System. Moreover, GA was shown to evidently decrease the activity of matrix metalloproteinases (MMP)-2 and MMP-9. The treatment led to decreased levels of several proteins in SCC-4 cells, including MEKK3, FAK, p-PERK, p-ERK1/2, p-p38, p-JNK1/2, RhoA, SOS1, p-AKT(Thr308), Ras, PKC, PI3K, NF-κB p65, as well as MMP-2 and MMP-9. Furthermore, GA was effective in diminishing the translocation of NF-κB and RhoA from the cytosol to the nucleus in SCC-4 cells. Overall, GA inhibits the migration and invasion of SCC-4 cells mainly by suppressing NF-κB expression, which in turn leads to a reduction in MMP-2 and MMP-9 activity [124].

The capability of GA in the modulation of anticancer effects of CPT in human breast adenocarcinoma cells was checked. In treated cells, a dose-dependent decrease in the breast cells’ viability at all treatment regimens was noticed. The CPT dose of required for 50% inhibition of growth of MCF-7 cells was found to be 10 μg/mL for 24 h. In the combination treatment groups, cellular death of 87.3% was detected in GA-treated (5 μg/mL) plus CPT-treated (10 μg/mL) cells. A noteworthy increase in cell survival was detected in GA-treated (0.5, 1.0, 2.5, 5.0, and 10 μg/mL) cells as compared to control untreated cells. MCF-10A cells treated with CPT exhibited 51.8% of cell death, demonstrating its cytotoxicity on normal cells. Moreover, the frequency of micronuclei (MN) was reduced in combinational therapy, probably decreasing the risk of chemotherapy-induced MN. Furthermore, GA in mono or combinational therapy did not cause any cytotoxic activities in normal breast epithelial cells. Also, GA did not display any substantial difference in colony inhibition when compared to CPT [125]. The effect of GA or cisplatin (CDDP) on cell viability was checked, using the non-small cell lung carcinoma (NSCLC) cell line A549 as well as normal human lung fibroblast cell line WI-26. Cell viability of both cell lines was inhibited in a dose-dependent manner when exposed to CDDP. GA exposure reduced cell viability of A549 from 50 to 200 μM in a dose-dependent manner. For A549 and WI-26 cells, the inhibitory concentration of 50 (IC50) values achieved for GA were 74.19 and 148.1 μM, respectively, and for CDDP were 12.13 and 15.80 μM, respectively. Furthermore, both GA and cisplatin were effective in preventing tumor spheroid formation and colony formation. Additionally, gallic acid and cisplatin prompted apoptosis. The anticancer effects of gallic acid were demonstrated in an in vivo mouse model [126].

The impact of GA on protein levels and gene expression of MMP-2 and MMP-9, as well as the in vitro migration and invasiveness of melanoma cells were examined. GA reduces the levels of MMPs and associated signaling pathway proteins, as well as MMP mRNA levels in A375.S2 human melanoma cells. The results indicate that GA possesses antimetastatic properties by diminishing the invasiveness of cancer cells. Furthermore, this effect of GA involved in the Ras and p-ERK signaling pathways, leading to the inhibition of MMP-2 in A375.S2 human melanoma cells [127]. The effects of GA on the proliferation as well as apoptosis of colon cancer cells was examined. HT29 and HCT116 cells were treated with numerous concentrations of GA for 24 h. The outcomes designated that GA promoted cell apoptosis through upregulating the ratio of cleaved caspase-3/pro-caspase-3 and cleaved caspase-9/pro-caspase-9 and inhibited cell proliferation. Moreover, GA reduced the level of phosphorylated (p)-SRC, p-STAT3p-EGFR, and p-AKT. In vivo, GA promoted tumor apoptosis, prevented tumor growth, and reduced the level of p-SRC, p-STAT3, p-EGFR, and p-AKT. It was concluded that GA was designated to suppress proliferation of colon cancer through SRC and EGFR phosphorylation inhibition [128]. Gallic acid was found to enhance the expression of Fas, FasL, and DR5 in gastric cancer cells. Furthermore, p53 was shown to play a role in the regulation of Fas, FasL, and DR5 expression induced by gallic acid [129]. The anticancer potential of GA was measured in three osteosarcoma cell lines: 143B, U2OS, and MG63. The GA impact on cell proliferation, apoptosis, cell cycle progression, and migration. Moreover, the in vivo antitumor activity of GA was observed using an orthotopic mouse model. Cell viability assays exhibited that GA meaningfully inhibited growth of 143B, MG63, and U2OS cells in a dose- and time-dependent manner at concentrations ranging from 50 to 200 µM. The IC_50_ value for GA was around 150 µM in all three cell lines after 48 h of treatment. GA suppressed tumor growth in vitro by inducing cell cycle arrest and apoptosis, and it also inhibited cell invasion and metastasis. Consistent with these findings, GA significantly reduced tumor growth in vivo in the orthotopic osteosarcoma model [130]. The impact of GA on the inhibition of proliferation and the induction of apoptosis in a lymphoblastic leukemia cell line was determined. Cell viability was about 93%, 87%, and 65% at a concentration of 20 μM after 24, 48, and 72 h, correspondingly. However, viability decreased to 13%, 8%, as well as 5% at a concentration of 100 μM after the above incubation periods. IC50 (inhibition concentration) was 60.3 ± 1.6 μM, 50.9 ± 1.5 μM as well as 30.9 ± 2.8 μM after a period of 24, 48, and 72 h, correspondingly. Remarkably, all tested concentrations of GA (10, 30, 50, and 80 μM) meaningly promoted apoptosis as compared to the control. These findings designate that the GA is effective in inhibiting proliferation and inducing apoptosis in Jurkat cells [131].

GA inhibited tumorigenesis both in vitro and in vivo via the MALAT1-Wnt/β-catenin signaling pathway, indicating its strong potential for development as a chemopreventive and therapeutic agent for patients with hepatocellular carcinoma (HCC) [132]. Treating human cancer cells (HeLa and HTB 35) with gallic acid significantly decreased cell viability in a dose-dependent manner. Gallic acid also markedly inhibited the proliferation of human cervical cancer cells and the formation of tubes in human umbilical vein endothelial cells. Additionally, it reduced the invasion of both cancer cells in vitro [133]. Gallic acid (GA) effectively inhibited the invasion and migration of human prostate cancer cells PC-3 cells in a dose-dependent manner. It also decreased the protein levels of MMP-2 and MMP-9. The findings suggested that GA exerts its effects by blocking the p38, PKC, JNK, and PI3K/AKT signaling pathways, along with reducing the levels of NF-κB protein. This modulation ultimately leads to the inhibition of MMP-2 and MMP-9 in human prostate cancer cells [134]. GA triggered apoptosis in human melanoma cells. It caused morphological changes and induced apoptosis in a dose- and time-dependent manner, resulting in a reduction in the percentage of viable cells. Moreover, GA down-regulated antiapoptotic proteins and up-regulated the proapoptotic proteins [135].

**Table 6:** Anticancer potential of gallic acid through different mechanisms. GA shows anticancer potential by reducing angiogenesis and induction of apoptosis and finally contributes to suppression of tumor progression.

	Cancer Types	Study Types	Animals/Cell Lines	Doses	Outcomes	Refs.
**Anticancer activity**	Oral	In vitro	SCC-4	0, 5, 30, 60 μM	° GA inhibited the adhesion of cancer cells ° GA suppressed the migration ° GA inhibited MMP-2 activity	[124]
	Breast	In vitro	MCF-7	2.5, 5.0, 10 μg/mL	° In combination therapy (gallic acid and Cisplatin), GA synergistically reduced the cancer cell viability	[125]
	Lung	In vitro	A549	75μM	° Formation of colonies and tumor spheroids was inhibited by treatment with GA	[126]
	Gastric	In vitro	AGS	5 μM	° GA induced apoptosis ° Gallic acid mediated apoptosis	[129]
	Bone	In vitro	143 B, MG63 and U2OS	150 μM	° GA inhibited the invasion and metastasis ° It induced cell cycle arrest as well as apoptosis	[130]
	Blood	In vitro	Jurkat cell (C121)	10, 30, 50, 80 μM	° GA treatment increased the % of apoptotic cells	[131]
	Liver	In vitro	HepG2 cells and Bel-7402	80, 120 μM	° GA (120 Μm) induced cell cycle arrest and apoptosis ° GA (80 μM) inhibited the EMT and metastasis	[132]
	Liver	In vivo	Xenograft Animal Model (HepG2 cells injected into nude BALB/c mice)	80 mg/kg	° GA reduced the tumor growth and induced the apoptosis	[132]
	Cervix	In vitro	HeLa and HTB-35	10, 12.5, 15 μg/mL	° GA inhibited proliferation of cancer cells and reduces invasion migration and	[133]
	Prostate	In vitro	PC3	25, 50, 100 µM	° GA inhibited cell invasion and migration ° GA inhibited the adhesion	[134]
	Skin	In vitro	A375.S2	0, 50, 100, 250, 300 μM	° GA induced cell cycle arrest and caused apoptotic cell death	[135]

### 4.9. Effect on Obesity

Obesity is a significant global health issue. The World Health Organization (WHO) defines overweight in adults as a body mass index (BMI) of 25–29.9 kg/m^2^, and obesity as a BMI of 30 kg/m^2^ or higher [136]. The high prevalence of obesity leads to significant health issues, increased mortality, and imposes a substantial economic burden on affected countries [137,138]. Numerous synthetic drugs and naturally occurring phytochemicals have been extensively researched for their antioxidant effects in addressing free radical generation associated with obesity, oxidative stress, and related complications [139,140]. The effects of GA on obesity were studied in a rat model with diet-induced obesity. Findings indicated that the liver weight, body weight, and adipose tissue weights in both peritoneal and epididymal areas were meaningfully lower in the HFD and GA groups as compared to the HFD group. Furthermore, serum levels of TAG, total cholesterol, phospholipids, LDL cholesterol, leptin, and insulin were also significantly reduced in the HFD and GA groups relative to the HFD group. Histological analysis revealed that the lipid droplets in rats on the HFD and GA diet were notably smaller than those in the HFD diet group. Furthermore, hepatic cholesterol and TAG levels were meaningfully reduced in the HFD and GA groups compared with the HFD group [141]. It was reported that triglyceride concentrations in the gallic acid group were improved as compared to the control group. In the epididymal white adipose tissue of the gallic acid group, there was a clear reduction in adipocyte size, activation of the Akt signaling pathway, as well as an increase in PPARγ expression. This finding proves that gallic acid improves lipid metabolism and glucose tolerance in obese mice [142]. Another study found that gallic acid (GA) meaningfully decreased the weight of perirenal adipose tissue in rats fed a high-fat diet (HFD). Additionally, GA improved the expression of proteins related to glycolysis and lipolysis. This research indicated that GA helps mitigate hypertriglyceridemia and fat accumulation by promoting lipolysis and glycolysis [143]. The potential effects of gallic acid (GA) in countering the impacts of long-term exposure to a high-fat diet (HFD) were explored. Administration of gallic acid resulted in lower serum levels of glucose, triglycerides, and cholesterol, while promoting an increase in high-density lipoprotein (HDL) levels. Additionally, the HFD group showed a reduced tolerance during a 2-h intraperitoneal glucose tolerance test. These results indicate that GA enhances the antioxidative profile in the hypothalamus and improves insulin sensitivity, as well as glucose and lipid metabolism, in the context of advancing obesity [144]. The anti-obesity effects of Chinese black tea, Pu-erh tea, and gallic acid (GA) were examined. Both Chinese black tea extract (BTE) as well as GA confirmed a dose-dependent inhibition of pancreatic lipase activity in vitro. Black tea extract and GA meaningfully suppressed the raise of blood triglyceride after oral administration of a corn oil emulsion to mice. Furthermore, the anti-obesity effects of GA and BTE were also assessed in a mouse model of diet-induced obesity. The GA 0.1% and BTE 0.6% groups exhibited substantial suppression of weight gain. These outcomes propose that GA contributes to the anti-obesity role of BTE as an active constituent via inhibiting pancreatic lipase activity [145]. Mice with diet-induced obesity that received GA treatment showed noteworthy improvements in glucose and insulin homeostasis. Furthermore, GA administration efficiently prevented weight gain without a change in food intake. Biochemical analyses exhibited a substantial activation of AMPK in the muscle, liver and interscapular brown adipose tissue of the mice treated with GA. Furthermore, levels of uncoupling protein 1 and other genes associated with energy expenditure were meaningfully increased in the interscapular brown adipose tissue. Collectively, these findings suggest that GA exerts its beneficial metabolic effects by activating the AMPK/Sirt1/PGC1α pathway and changing the interscapular brown adipose tissue genes associated to thermogenesis. This study emphasizes that targeting the activation of the AMPK/Sirt1/PGC1α pathway through GA or its derivatives may serve as a promising therapeutic approach for addressing insulin resistance in metabolic disorders [146].

### 4.10. Role in Osteoarthritis

The role of GA in the treatment of anti-synovial fibrosis and synovial inflammation in knee osteoarthritis was investigated. It was demonstrated that GA reduced the levels of IL-6, TNF-α, and IL-1β, and reduced the protein expression levels of TGF-β, α-SMA, as well as Collagen I in synovial tissues and cells. Moreover, the therapeutic effect of GA on knee osteoarthritis, fibrosis, and synovitis is moderately attributed to the mitigation of metabolic disorders and the rebalancing of the intestinal flora [147]. An in vivo study showed that GA reduced cartilage degradation compared with the vehicle group. The study findings established the chondroprotective role of GA and provided a possible drug for the relief of osteoarthritis [148]. The effects of GA and mechanical stretching on the expression of anabolic as well as catabolic genes and restoring ECM production by osteoarthritic human articular chondrocytes (hAChs) cultured in monolayers were investigated. It was reported that CTS as well as GA acted synergistically to promote the deposition of collagen and glycosaminoglycan in the ECM by fourteen- as well as seven-fold, correspondingly. Further, the synergistic stimuli selectively upregulated the expression of cartilage-specific proteins, COL2A1 by 47 times and COL11A2 by 7 times, and, in contrast, downregulated the expression of MMP-13 by 125 times and MMP-1 by 2.5-fold [149].

### 4.11. Anti-Colitis Potential

Inflammatory bowel disease (IBD), which includes ulcerative colitis (UC), Crohn’s disease (CD), and IBD unclassified (IBDU), is a group of chronic or recurrent inflammatory disorders that primarily affect the small and large intestines [150]. The current treatment of inflammatory bowel disease, including anti-inflammatory drugs and antibiotics, is effective but also causes adverse effects. In this regard, natural compounds, or bioactive compounds, have been shown to play a role through various mechanisms. Anti-colitis potential of gallic acid through different mechanisms is described in Table 7. GA attenuated weight loss, comforted symptoms, and ameliorated colonic morphological injury in mice with DSS-induced colitis [151]. A substantial reduction of clinical symptoms and weight loss was detected in dextran sodium sulfate-exposed, GA-treated mice in comparison to control mice. This effect is associated with a significant amelioration of colonic architectural disruption, and a meaningful decrease in colonic myeloperoxidase activity [152]. GA reduced the disease activity index, histopathological evidence of injury, and colon shortening. Additionally, GA upregulates the expression of Nrf2 and its downstream targets in DSS-induced colitis in mice [153]. Another study result demonstrated that tissue MPO, MDA, Cathepsin B, and Cathepsin L values increased in the colitis group. However, Cathepsin B, Cathepsin L, MDA, and MPO values exhibited a considerable decrease in animals with GA administration in TNBS-induced colitis. Treatment by GA diminished inflammatory cell infiltration [154]. GA treatment improved the systemic inflammatory response, colitis symptoms, and reliance on gut microbiota, as demonstrated by microbiota depletion as well as fecal microbiota transplantation. Moreover, GA upregulated the biosynthesis of secondary bile acids and altered the gut microbiota community structure. Moreover, this study explained that GA efficiently mitigates colitis via modulating the Treg/Th17 balance, facilitated through the enhanced synthesis of microbiota-derived SBAs [155].

### 4.12. Role in Respiratory System

**Figure 7:**
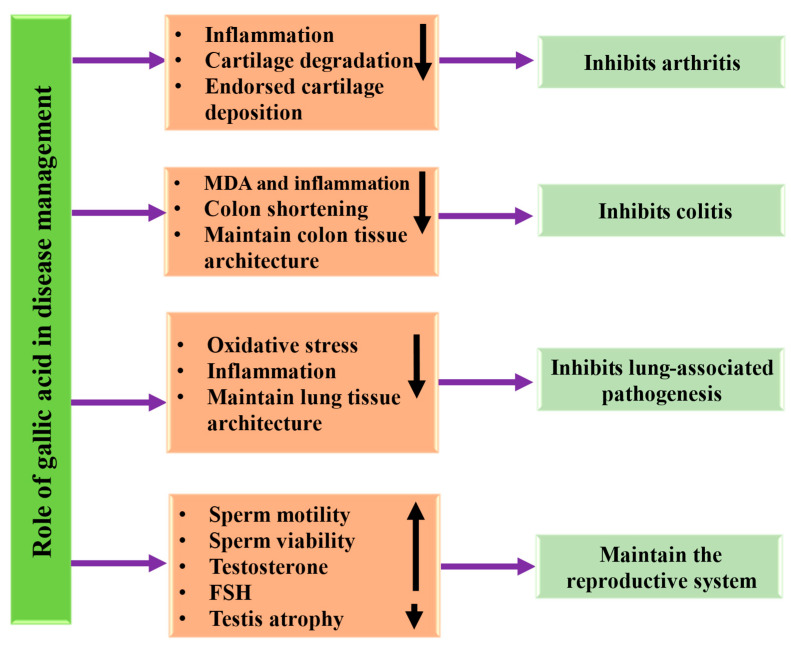
The role of GA in different diseases. GA shows broad therapeutic potential across multiple diseases via cytoprotective, maintenance of tissues integrity, and finally management of various pathogenesis. An upward arrow (↑) denotes increased, and a downward arrow (↓) denotes decreased.

The potential role of gallic acid is attributed to several mechanisms that help protect lung tissues from damage. The potential role of gallic acid in the respiratory system through different mechanisms is described in Table 7 and Figure 7. The role of GA against COPD-associated lung inflammation/emphysema was checked. GA suppressed the ET-induced neutrophil infiltration and elevated MPO activity. Additionally, GA provided protection against ET-induced airspace enlargement and ameliorated MMP-2/MMP-9 [156]. The study examined the effectiveness of GA in treating asthma and explored its mechanisms. It was found that ovalbumin significantly increased infiltration of pro-inflammatory cells, including lymphocytes, eosinophils, neutrophils, and macrophages, which was associated with enhanced airway hyperresponsiveness. In contrast, GA was shown to decrease the infiltration of these pro-inflammatory cells and improve airway hyperresponsiveness. Additionally, GA reduced IL-13 and IL-5 levels in bronchoalveolar lavage fluid (BALF) and decreased IL-33 expression in lung tissues [58]. A study result reported that gallic acid demonstrated the ability to alleviate airway hyperresponsiveness and decrease the infiltration of pro-inflammatory cells. Overall, this result concluded that gallic acid alleviates ovalbumin-induced asthma, probably by preventing IL-33-mediated ILC2 activation and subsequent Th2 cytokine release through downregulation of the MyD88/NF-κB signaling pathway [58]. The potential role of gallic acid in the inflammatory process and the probable signaling pathway in elastase-induced emphysema was studied. The porcine pancreatic elastase (PPE) group exhibited pulmonary edema and a noteworthy change in arterial blood gas values as compared to the control group. Co-treatment with gallic acid well-preserved all such changes almost to the normal levels [44]. A study showed that intratracheal Bleomycin (BLM) administration increased collagen content, inflammatory or fibrotic changes, pro-inflammatory cytokines, and malondialdehyde levels in the lung. However, GA oral administration reversed all of these histopathological alterations and biochemical indices induced by BLM [157].

### 4.13. Role in Reproductive System

The complication related to the reproductive system is a significant problem. It was reported that natural products play a significant role in the reproductive system due to their safety and lower cost. The potential role of gallic acid in reproductive systems through different mechanisms is described in Table 7 and Figure 7. The impact of GA and metformin was examined regarding follicle-stimulating hormones, testosterone, luteinizing hormone, sperm count, and the histological alterations in the testes of diabetic mice. In the methylglyoxal (MGO) group, testosterone levels, testis volume, sperm count, diameters of seminiferous tubules, epithelial height, and superoxide dismutase activity were all found to be decreased as compared to the control group. The findings advocate that gallic acid and metformin helped to counteract the effects of methylglyoxal [165]. Another study intended to explore the impact of gallic acid on the ovarian pathophysiology in an animal model of polycystic ovary syndrome (PCOS). The LETZ-treated animals exhibited PCOS-like symptoms, including increased body and ovarian weight, elevated serum testosterone levels, a higher LH/FSH ratio, elevated inflammatory cytokines, reduced serum estrogen, and changes in ovarian cytoarchitecture. Also, LETZ-induced PCOS, when treated with gallic acid, was observed as a reduction in testosterone and LH/FSH ratio [158].

The roles of GA against testis as well as epididymis toxicity were studied. It showed that, compared with the control group, total seminiferous tubule volume and total testicular volume decreased in the CP group. Moreover, compared with the cisplatin group, a significant increase was noticed in the total testicular volume and total seminiferous tubule volume in the CP and GA group. The height of germinal epithelium decreased in the CP group, and an increase was detected in the CP and GA group as compared with the CP group. Additionally, gallic acid improves cisplatin-induced male reproductive toxicity by restoring structural and functional deterioration [159]. In the epididymis and testes of the rats treated with cyclophosphamide (CPA), there was an increase in malondialdehyde, hydrogen peroxide, and nitrite levels. Concurrently, plasma levels of LH, FSH, and testosterone significantly decreased, leading to reduced sperm motility, viability, and notable atrophy of the testes and epididymis in CPA-treated rats. However, gallic acid (GA) was able to reverse these alterations [166]. In the CP group, an increase in the sperm DNA compared with the control group was noticed, and it was improved in the GA and CP group compared to the CP group. Moreover, an increase in MDA levels in the CP group was detected, but a decrease in the GA and CP group. The histopathological finding revealed obvious testicular atrophy in the CP group, while GA reduced these deviations [167].

### 4.14. Wound Healing Effects

Gallic acid enhances the expression of antioxidant genes and promotes the migration of fibroblasts and keratinocytes. Additionally, factors activated by GA treatment are known to be key components of wound healing, such as extracellular signal-regulated kinases, c-Jun N-terminal kinases, and focal adhesion kinases, thereby highlighting the role of gallic acid in the wound repair process [168]. The objective of this study is to propose a gallic acid (GA)- functionalized silk fibroin (SF) and gelatin (Gel) composite wound dressing, in which gallic acid is used as a wound-healing and antibacterial agent. The result was reported as a cutaneous excisional mouse wound model, evidencing the effective ability of SF-Gel-GA to promote wound healing. Compared to pure SF dressing and commercial Tegaderm Hydrocolloid3M dressing, the wound closure rate by SF-Gel-GA treatment was meaningfully improved. The histological findings further prove that SF-Gel-GA facilitates collagen deposition, epithelialization, and neovascularization at wound sites to promote wound healing [169]. Another finding showed that diabetic wounds treated with the GA-CSNPs nanocomposite scaffold demonstrated a faster rate of wound closure; histopathology revealed reduced inflammatory cells, accelerated fibroblast migration, and enhanced collagen, along with increased hexosamine synthesis [170]. It was noticed that MDA and MPO levels increased while GSH, epithelization, FGF, and EGF expression levels were decreased. Gallic acid treatment showed a role in the improvement of these scores. Degenerated gingival epithelium, connective tissue fibers, and disintegration in epithelial, edema, and inflammatory cells were noticed in the burn group. Moreover, gallic acid treatment after the burn improved the pathological changes [160].

### 4.15. Antihypertensive Effects

Antihypertensive potential of gallic acid through different mechanisms is described in Table 7. The antihypertensive and vasorelaxant effects of GA from the green alga Spirogyra sp. were studied. Its antihypertensive effect was examined via investigative GA-mediated inhibition of the angiotensin-I converting enzyme. In addition, GA significantly reduced blood pressure in spontaneously hypertensive rats, effects similar to those of captopril [171]. To study the functional effects of GA on blood pressure regulation in Ang II-infused mice, wild-type mice were treated with varying doses of GA (5 or 20 mg/kg body weight) and infused with Ang II (490 ng/kg/min). Additionally, Ang II-induced increases in aortic wall thickening, collagen deposition, and the accumulation of Mac-2-positive macrophages were also blunted in GA-treated mice [161].

### 4.16. Radioprotective Effects

The radioprotective potential of gallic acid from Euphorbia hirta against gamma irradiation-induced radiotoxicity in human lymphocytes was evaluated. The frequency of micronuclei significantly declined when cells were preprocessed with gallic acid before being exposed to 2 Gy gamma radiation. In the same way, pre-gamma radiation treatment of human cells by gallic acid led to evidently less DNA injury [162]. Oral administration of GA one hour before whole body gamma radiation exposure reduced the radiation-caused cellular DNA damage in mouse peripheral blood leukocytes, splenocytes, and bone marrow cells. The GA administration prevented radiation-caused mortality as well as weight loss in animals exposed to a lethal dose of gamma radiation [163]. Gallic acid reduces DNA and oxidative damage, inhibits the apoptosis of bone marrow cells, and reduces bone marrow cell cycle arrest in radiation-damaged mice. It has radioprotective effects on these cells [172].

### 4.17. Role in Eye Health

The potential role of GA in eye health is attributed to several mechanisms that help protect the eyes from complications. The potential role of gallic acid in eye health through different mechanisms is described in Table 7. GA significantly reduced intracellular reactive oxygen species (ROS) in lipopolysaccharide (LPS)-activated macrophages and human corneal epithelial cells (HCECs) exposed to hyperosmotic stress. In vivo efficacy testing conducted in a mouse model of Experimental Dry Eye demonstrated that GA effectively prevents and inhibits the apoptosis of corneal epithelial cells. Additionally, it reduces inflammatory factors in both the cornea and the conjunctiva while protecting goblet cells [173]. The effect of GA and its combination with glibenclamide on biochemical markers and the histology of the cornea in streptozotocin (STZ) induced diabetic rats was explored. The administration of STZ led to the development of insulin resistance, hyperglycemia, weight loss, microproteinuria, oxidative stress, inflammation, as well as alterations in corneal histology. However, these adverse effects were mitigated after the rats received supplementation with GA alone or in combination with glibenclamide [164]. GA has demonstrated antiretinal angiogenic activity by inhibiting the phosphorylation of p38, ERK, and NF-κB, and by reducing the expression of inflammatory cytokines [174].

### 4.18. Role in Oral Health

A study investigated the role of a mouth spray containing gallic acid on the oral microbiota as well as dental health of healthy cats. Findings indicated that after forty-two days of treatment, there was an improvement in both plaque and gingival indexes. The mouth spray not only reduced harmful bacteria but also promoted the growth of healthy oral microbiota. This initial research suggests that a gallic acid mouth spray could be a valuable product for improving oral hygiene in cats [175]. The study assessed the effectiveness of a formulated GA varnish for treating enamel caries lesions. The 0.5% GA varnish showed the highest surface microhardness recovery (SMHR%) across all depths; however, the substantial difference from other experimental groups was observed at a depth of 30 μm. The SMHR% for fluoride, as well as the 2% and 8% GA varnishes, was comparable at all depths. All treatments can potentially re-mineralize enamel lesions, with 0.5% GA varnish showing the most noteworthy effect, specifically on the surface layer [176].

The research focused on assessing the impact of gallic acid (GA) combined with sodium fluoride (NaF) on dentinal tubules in vitro. Both NaF and the combination of GA and NaF significantly decreased dentin permeability. Remarkably, the GA and NaF treatment exhibited a more noticeable effect. Following exposure to acid challenges, both treatment groups demonstrated lower dentin permeability compared to the initial evaluations. The study concluded that the combined effect of GA and NaF is more effective than NaF alone in reducing dentin permeability [177].

### 4.19. Antimicrobial Activity

**Figure 8:**
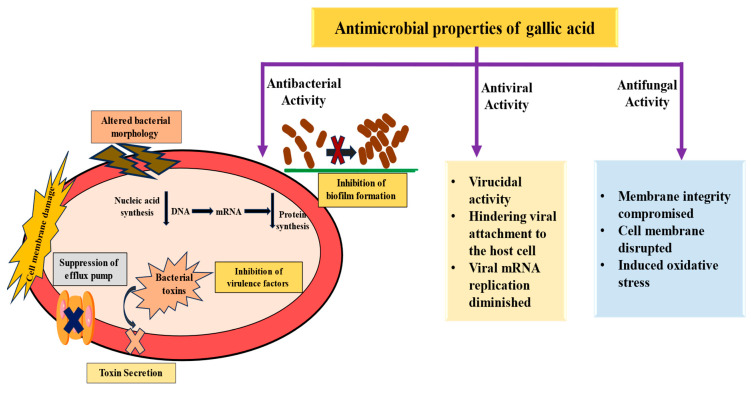
Antimicrobial activity of GA through different mechanisms. GA shows broad-spectrum antimicrobial effects through the disruption of microbial cell membranes, inhibition of biofilm formation, and interference with essential metabolic pathways. Collectively, these actions contribute to the powerful antimicrobial potential of GA. A downward arrow (↓) denotes decreased.

The problem of antibiotic resistance, especially among Gram-negative strains, is increasingly concerning, particularly in hospital settings where at-risk patients are more susceptible [178]. Plant extracts derived from various parts, such as roots, stems, fruits, and flowers, are frequently utilized to suppress the growth of microorganisms [179]. Gallic acid has shown substantial antimicrobial potential, suggesting a possible role in hindering disease initiation as well as progression. The implication of GA in the inhibition of microorganisms is presented in Table 8 and Figure 8.

#### 4.19.1. Antibacterial Activity

Gallic acid and methyl gallate were noted to exhibit antibacterial activity against bacterial strains at concentrations between 64 and 256 µg/mL. Both substances effectively diminished bacterial growth as well as metabolic activity of the strains [180]. Furthermore, gallic acid exhibited bactericidal activity and inhibited the formation of bacterial biofilm. Moreover, GA demonstrated protective effects against bacterial infections and enhanced the survival rates of BALB/c mice and Galleria mellonella [181]. Research has demonstrated that gallic acid (GA) possesses antimicrobial properties against a range of bacteria and also enhances the effectiveness of antibacterial agents like ciprofloxacin, norfloxacin, erythromycin, penicillin, ampicillin, oxacillin, gentamicin, and through a synergistic mechanism [182]. Another study examined the antibacterial effects of caffeic acid, GA, ellagic acid, kaempferol, quercetin, and rutin on skin infections caused by Staphylococcus epidermidis, Staphylococcus aureus, and Klebsiella pneumoniae. The findings revealed that GA exhibited stronger antibacterial activity against these bacteria compared to the other compounds tested [183]. The values for Minimum Inhibitory Concentration (MIC) and Minimum Bactericidal Concentration (MBC) of gallic acid (GA) against planktonic Sh. flexneri were found to be 2 mg/mL and 8 mg/mL, respectively. Additionally, varying the concentration and duration of treatment with gallic acid demonstrated its inhibitory effects, evidenced by decreased cell viability, damaged cell membranes, and alterations in bacterial morphology. GA was found to meaningfully inhibit the formation of biofilms by Shigella flexneri, as it penetrated into the EPS and decreased the count of viable bacteria [184]. Quantitative staining of biofilms with crystal violet demonstrated that gallic acid at a concentration of 2 mg/mL can significantly inhibit biofilm formation. Analysis of viable bacteria within the biofilm indicated that gallic acid was able to penetrate and eliminate S. aureus [185]. It also studied the effect of gallic acid on the *ica* family gene expression and polysaccharide slime formation in *S. aureus* biofilm formation. The results exhibited that *icaR* was meaningfully activated, while *icaA* and *icaD* were downregulated in a dose-dependent way of increasing concentrations of GA [185].

#### 4.19.2. Antiviral Activity

Jadel Müller Kratz and colleagues (2008) [186] demonstrated that replication of HSV-2gallic was prevented by treatment of gallic acid in a concentration-dependent way. This inhibition occurred when the compound was incubated with the virus before introducing the mixture to cells, as well as when it was added to cells after infection. Furthermore, gallic acid was noted for its virucidal activity against HSV-2 [186]. Another study revealed that gallic acid treatment significantly decreased the production of virulent H1N1 influenza virus proteins. Additionally, regarding the autophagic mechanism, there was a notable reduction in both the conversion of LC3B II and the ratio of LC3B II to LC3B I levels. The acridine orange staining assay demonstrated a decrease in the accumulation of autophagosomes in cells infected with the H1N1 influenza virus [187]. A study checked whether gallic acid (GA) has anti-HCV activity. GA downregulated the expression levels of HCV-RNA (~50%) and NS5A-HCV proteins (~55%) in a time-dependent way [188]. A different study designated that gallic acid effectively inhibits the replication of influenza virus mRNA as well as the development of MDCK plaques [189]. It was reported that gallic acid inhibited the proliferation of HPVep cells and showed important specificity towards HPV-positive cells. These outcomes suggest that gallic acid is a promising candidate for the development of anti-HPV agents [190]. An antiviral assay showed that GA exhibited strong antiviral activity against various viruses, without causing cytotoxicity at the tested concentration [191].

#### 4.19.3. Antifungal Activity

An experiment was performed to recognize an antifungal compound from *Sarcochlamys pulcherrima*, that can prevent the growth of *Candida auris*. The in vitro antifungal test demonstrated that gallic acid was effective in inhibiting the growth of various C. auris strains [192]. Another study demonstrated that GA possesses a broad range of antifungal activity. The in vivo model showed that administering GA via intraperitoneal injection at a dose of 80 mg/kg per day meaningfully improved the cure rate in a mouse model of systemic fungal infection [193]. The antifungal effects of GA against fluconazole-resistant strains of Candida spp. were investigated. GA exhibited minimum inhibitory concentrations between 16 and 72 μg/mL, leading to changes in mitochondrial transmembrane potential, and disruption of membrane integrity [194]. The antifungal activity of methanolic extracts from the bark of Terminalia nigrovenulosa (TNB) was investigated. The purified antifungal compound was gallic acid and it showed high antifungal activity against F. solani. After 24 h of incubation with GA (500 ppm), the hyphae became collapsed and shrunken [195]. The study aimed to investigate the antifungal and anti-inflammatory properties of gallic acid (GA) on keratitis caused by Aspergillus fumigatus. In vitro, GA meaningly inhibited the growth of A. fumigatus, as well as its biofilm formation and adhesion. In vivo, a concentration of 100 µg/mL GA alleviated the severity of fungal keratitis by dropping fungal load, lowering MPO activity and reducing neutrophil infiltration [196].

## 5. Synergistic Effects of Gallic Acid with Other Compound/Drugs

**Figure 9:**
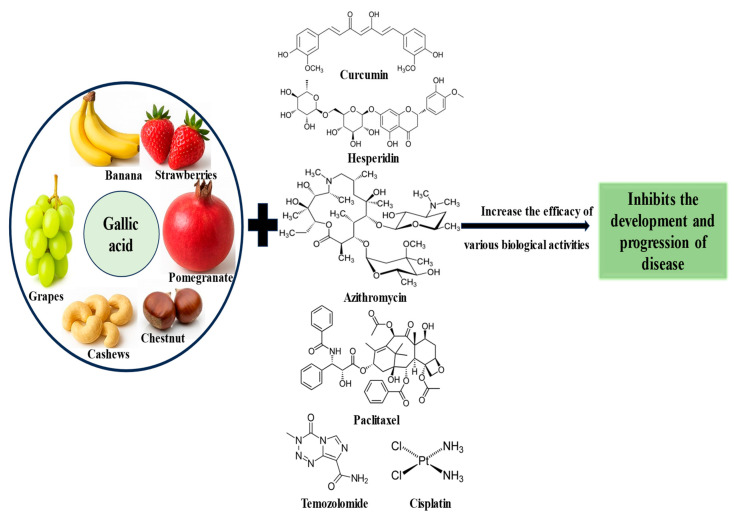
Synergistic effects of gallic acid with other compound/drugs. GA enhances the therapeutic potential of other natural compounds/drugs through synergistic interactions. These synergistic activities involve improvement through anticancer activity, increased drug uptake, and modulation of signaling pathways.

The combined use of gallic acid with other bioactive compounds or drugs exhibits synergistic effects by enhancing overall therapeutic actions against pathogenesis through different mechanisms [Table 9 and Figure 9]. Furthermore, the combination of drugs helps to reduce side effects and decrease drug resistance. The synergistic effects of gallic acid with other drugs have been reported in various pathogenesis. Some studies regarding the synergistic effects of gallic acid with other natural compounds and drugs are described here. Hassan Moghtaderi et al. (2018) [197] reported that the combination of gallic acid (GA) and curcumin (Cur) significantly reduced the growth of MDA-MB-231 breast cancer cells. Furthermore, this combination elevated reactive oxygen species (ROS) levels and boosted cytotoxicity, while reducing glutathione in MDA-MB-231 cells [197]. Another study outcome reported that curcumin revealed synergistic antioxidant potential when combined with GA whereas the antagonistic effect occurred in curcumin combination with xanthone or ascorbic acid [198]. The therapeutic effect of gallic acid (GA) as a potent antioxidant against the encysted phase of Trichinella spiralis was analyzed, both alone and in combination with albendazole (ALB). The study aimed to detect their synergistic effects on the histology and ultrastructure of cardiac and skeletal muscles. Findings exhibited that the decreased *Trichinella* sp. larvae g^−1^ in muscles of the group treated with the combination of GA as well as ALB exhibited overall decrease percentages. Administering both ALB and GA resulted in the greatest reduction in infections, along with improvements in biochemical markers and the restoration of histological and ultrastructural features to a normal state [199]. The therapeutic mechanism of a combination of gallic acid and hesperidin against colorectal cancer was studied. The combined extract treatment demonstrated a greater reduction in CRC cell viability than either gallic acid or hesperidin used individually [200]. Another study exhibited that the combination of AZM/GA had an additive effect against P. aeruginosa and MSSA and a synergistic effect against MRSA [201].

The anticancer activity of gallic acid, both alone and in combination with paclitaxel and/or carboplatin, was assessed. It was found that gallic acid, paclitaxel, as well as carboplatin, whether administered individually or in combination, efficiently ceased cell cycle progression at the G2/M phase and triggered pre-G1 apoptosis. Additionally, the triplet combination significantly increased the mRNA expression levels of Bax, P53, and CASP-3 in MCF-7 cells compared to single or combined treatments [202]. Another study result reported that, compared to the action of TMZ (Temozolomide) alone or GA alone, the TMZ/Ga combination increased the inhibition of cellular viability and apoptotic level in the glioma cell line [203]. Another study based on lung cancer reported that gallic acid combined with cisplatin showed synergistic effects on inducing the pro-apoptotic mediators as well as modulating the activation of apoptosis-related molecules [204]. The ability of gallic acid (GA) to modulate the anticancer potential of Cisplatin (CPT) in human breast adenocarcinoma cells (MCF-7) was examined. GA and CPT showed important cytotoxic activities in MCF-7 cells in a dose-dependent way. In combination therapy, GA synergistically reduced the MCF-7 cell viability compared to the individual therapies [125]. Gallic acid (GA) increased the anticancer role of cisplatin in inhibiting cancer cell proliferation and inducing cell apoptosis following elevated Bax expression and suppressed expression of Bcl-2. Additionally, the study’s results also established that GA showed independent anticancer effects on lung cancer A549 cells, facilitating cisplatin’s anticancer effects [205]. A study focusing on leukemia cancer cells found that the combination of gallic acid (GA) and Pirarubicin (Pira) meaningfully diminished cell viability, mitochondrial membrane potential, as well as mitochondrial activity in K562 and K562/Dox cancer cells, in a GA concentration-dependent manner in comparison to untreated or Pira-treated cells [206]. Pretreatment with gallic acid as well as famotidine in combinations shows a role in the protection of the gastric mucosa, which was established by reduction in gastric juice volume, ulcer index, and free as well as total acidity [207].

## 6. Gallic Acid Based Nanoformulation and Its Application in Different Pathogenesis

Nanotechnology holds significant promise across numerous medical fields, including pharmaceutical research and drug development, cancer treatment, personalized medicine, vaccine innovation, disease diagnostics, and medical device and procedure advancements [208]. Nanoformulation activates the therapeutic potential of drugs with low oral bioavailability and offers several benefits, including enhanced drug solubility and bioavailability, reduced side effects, and longer circulation duration [209]. Nanotechnology-based delivery systems might improve the efficiency of dietary compounds, such as phenolic compounds [210,211,212]. Different types of nano formulation based on gallic acid have been synthesized, and their higher efficacies are reported [Table 10]. The gallic acid–phospholipid complex was prepared to examine the role of phospholipid complexation on oxidative damage induced by carbon tetrachloride (CCl4) in rat liver. This complex effectively lowered the levels of hepatic marker enzymes compared to the CCl4-treated group. Additionally, it enhanced the pharmacokinetics of gallic acid (GA) by improving its relative bioavailability and extending its elimination half-life. Therefore, the study designates that phospholipid complexation has augmented the therapeutic effectiveness of GA, likely due to improved absorption and increased bioavailability in rat serum [213].

Stealth liposomes were developed to deliver GA for the treatment of Alzheimer’s disease (AD). These liposomes were functionalized with transferrin (Tf) to enhance their targeting to the brain. The GA-loaded Tf-functionalized liposomes had an average diameter of 130 nm and displayed a low polydispersity index. Additionally, these nanocarriers facilitated sustained GA release over 5 days and demonstrated physical stability for 1 month under appropriate storage conditions. Notably, the GA-loaded Tf-functionalized liposomes exhibited a strong affinity for interacting with Aβ1-42 monomers, effectively slowing down both the conversion of monomers to oligomers and oligomers to fibrils, resulting in a 56% reduction in fibril formation. Moreover, the nanoparticles disaggregated approximately 30% of preformed Aβ fibrils. These findings suggest that Tf-functionalized liposomes may serve as an effective platform for delivering GA to the brain in the AD therapy [214]. Gallic acid powder, as well as liposome, meaningfully improve bone regeneration in rats with calvarial defects. The improvement in healing is obvious with increased OPG as well as BMP-2 expressions and reduced inflammation and RANKL expressions [215]. The study examined the protective role of pure gallic acid and gallic acid nanoparticles on nephrotoxicity. The mean particle sizes of nano-GA and Eudragit RS 100 nanoparticles were 180 ± 16.3 nm and 395.52 ± 1.68 nm, respectively. The nanoparticles exhibited a polydispersity index of 0.24 ± 0.02, and the distribution of the particles was monodisperse with homogeneous size distribution. The mean loading efficiency of nano-GA was 75.65 ± 7.35%, and the mean production yield was 83.35 ± 7.25%. Although the formulation demonstrated relatively low trapping efficiency, it is compatible with GA’s aqueous solubility. Study findings indicated that both gallic acid and nano-gallic acid reduced mitochondrial malondialdehyde levels, mitochondrial membrane damage, interleukin-6, TNF-α, and mitochondrial ROS production. Additionally, it significantly enhanced the levels of mitochondrial glutathione, superoxide dismutase, and catalase in comparison to the group treated with cisplatin. Histopathological analysis supported the results of the biochemical tests. This demonstrated that pure gallic acid, along with its nanoparticle form, alleviated renal oxidative stress, mitochondrial dysfunction, and inflammation associated with cisplatin-induced nephrotoxicity [216]. Another study effectively developed a nanoparticle (SC@Se/GA) that holds anti-inflammatory properties. The SC@Se/GA has a smaller size, better stability, as well as kidney-targeting. GPx enzyme activity of SC@Se/GA increases by nearly 50% more than SC@Se only, demonstrating its effective capability to scavenge ROS. Meanwhile, SC@Se/GA has a longer renal retention period than free drug gallic acid (GA), which can intensely decrease the levels of inflammatory factors. SC@Se/GA, through its synergistic antioxidant and inflammatory effects, noticeably alleviates cisplatin (CDDP)-induced renal injury as well as restores renal function [217].

Gallic acid was successfully encapsulated within poly (D, L-lactide-co-glycolide) (PLGA) nanoparticles, achieving an encapsulation efficiency of 82.86%, whereas gallic acid exhibited 98.24% in vitro release at 48 hours. Notably, gallic acid-loaded PLGA nanoparticles exhibited approximately 90% inhibitory activity against trophozoites. Furthermore, nano-encapsulation significantly reduced cytotoxicity toward MRC-5 cells compared with gallic acid [218]. Gallic acid-loaded graphene oxide-based nanoformulation (GAGO) was prepared, and the antibacterial activity of GAGO was evaluated. GA-loaded graphene oxide-based nanoformulation (GAGO) has the ability to load GA at 76.08 ± 6.823%, resulting in a GA loading of 0.942 ± 0.002 g/g of GAGO. The antibacterial activity of GA against MRSA enhanced meaningfully at lower concentrations as loaded onto GAGO nanoformulation [219]. Gallic acid (GA) has been shown to inhibit cancer cell proliferation by inducing apoptosis. To enhance the anticancer effects, gold nanoparticles (GNPs) were employed as a delivery system for gallic acid to target cancer cells. Interestingly, the GNPs–GA complex had a reduced ability compared to unmodified GA to prevent the growth of cervical cancer. Notably, at high concentrations (150 μM), the GNPs–GA complex exhibited no toxicity to normal cells, whereas GA on its own was cytotoxic [220]. The synthesized magnetite nanocomposite (Fe_3_O_4_–PEG–GA) demonstrated a substantially higher gallic acid loading capacity (35%). The in vitro release in human physiological pH 7.4 as well as pH 4.8 at 37 °C was made and was found to be more sustained for up to 8 days. The designed formulation exhibited better anticancer activity than free GA [221]. Silver nanoparticles synthesized using gallic acid in bentonite/starch bio-nanocomposites and its activity were determined. The formation of AgNPs was established by a UV-vis absorbance peak shown at 412 nm. AgNPs exhibited noteworthy entrapment efficiency (EE) with 80 ± 0.25%. The synthesized AgNPs in BNCs is a probable candidate for hindering the growth of pathogenic bacteria and exhibited noteworthy cytotoxicity against MCF-7 cancer cell line [222]. The complex gold nanoparticles-GA showed an improved capability to inhibit the proliferation of cancer cells as compared to GA alone, demonstrating that the anticancer effects of gallic acid can be enhanced by conjugation with gold nanoparticles [223].

The therapeutic use of gallic acid is constrained by various biological barriers, such as low solubility in water, quick degradation, limited systemic bioavailability, and difficulties in penetrating tissues, particularly the blood-brain barrier (BBB) in the context of neuroprotection. To overcome these limitations, nano-carrier systems have been specifically designed to modify their physicochemical characteristics in order to navigate these biological challenges effectively. These advantages include a higher drug-loading capacity, reduced required dosage and dosing frequency, and favorable biocompatibility, which collectively contribute to a lower adverse effect [224,225]. In addition, such systems enhance drug stability, bioavailability, and biodegradability. They also contribute to minimize the acquired drug resistance, reduce toxicity and immunogenicity, limit off-target effects, and compatibility with various administration routes, for example, intranasal, oral, intravenous, as well as intramuscular [226,227]. Nanocarriers provide opportunities to cross the BBB for treatment and diagnosis of brain diseases, including tumors. Several transport systems as carriers on brain endothelial cells deliver vital nutrients as well as endogenous substances to the brain, distinguished by their substrate specificity. These carriers comprise nutrient transporters such as glucose transporters (GLUT), peptide transporters, amino acid transporters, ion transporters, choline transporters, and so on [228]. The GA conjugated gold nanoparticles (GA-GNPs) were prepared by loading gallic acid onto GNPs. The GA–GNPs showed a substantial decrease in the survival of U251 glioblastoma cells as compared to GNPs alone as well as improved cell death induced by radiation. Furthermore, the combination of GA–GNPs as well as radiation directed to the arrest of the U251 cell cycle in the S and G2/M phases whereas apoptosis triggered, as demonstrated by decreased expression of BCL-2 and increased levels of BAX protein [229]. Gallic acid-loaded chitosan nanoparticles treatment improved the DNA damage by increased p-H2AX, PARP1, and suppressed the FEN-1 expression in MDA-MB-231 cells [230].

**Table 10:** Gallic acid based nanoformulation and its application in different pathogenesis. This table summarizes different nanoformulation strategies incorporating GA and its therapeutic applications in various diseases based on animal model and cell lines.

Formulation	Study Types	Role in Disease/Activity	Outcome	Refs.
GA-loaded transferrin (Tf)-functionalized liposomes	In vitro	Alzheimer’s disease	° GA-loaded Tf-functionalized liposomes showed a strong capability to interact with Aβ1-42 monomers, and reduced the number of fibrils	[214]
Gallic acid liposome	In vivo	Bone wound healing	° Gallic acid powder and liposome improved bone regeneration of calvarial defects rats	[215]
Gallic acid-loaded Eudragit-RS 100 nanoparticles	In vivo	Nephrotoxicity	° Formulation improved renal oxidative stress, mitochondrial dysfunction, and inflammation in nephrotoxicity induced by cisplatin	[216]
Gallic acid-selenium nanoparticles	In vivo	Renal injury	° This formulation alleviated renal injury and restores renal function	[217]
Gallic acid encapsulated in the PLGA nanoparticles	In vitro	Anti-acanthamoeba potential	° This formulation showed 90% inhibition against trophozoites.	[218]
Gallic-acid-loaded graphene-oxide-based nanoformulation	In vitro	Antibacterial	° Antibacterial agent against multi-drug-resistant bacteria	[219]
Gallic acid gold nanoparticle	In vitro	Anticancer	° Formulation inhibited the growth of cervical cancer cells	[220]
Fe_3_O_4_-PEG-GA	In vitro	Anticancer	° Improved anticancer action than free gallic acid	[221]
Gallic acid silver nanoparticle	In vitro	Antibacterial and anticancer	° Formulation inhibited pathogenic bacteria growth ° It exhibited cytotoxicity against cancer cell line	[222]
Gallic acid gold nanoparticle	In vitro	Anticancer	° Formulation inhibited the proliferation of cancer cells effectively	[223]
Gallic acid–gold nanoparticles	In vitro	Anticancer	° Survival of cancer cells inhibited by formulation ° Improved radiation-induced cell death ° Arrested the cell and activated apoptotic cell death	[229]
Gallic-acid-loaded chitosan nanoparticles	In vitro	Anticancer	° Formulation inhibited the expression of tumor proliferating markers ° Induced proapoptotic proteins	[230]

## 7. Toxicological Profile and Challenges in the Clinical Translation of Gallic Acid

The measurement of a drug or natural compound’s toxicity and safety profile is necessary for its use in disease management. Even compounds with therapeutic uses can cause adverse effects when used at inappropriate doses. Therefore, a detailed understanding of the beneficial and harmful effects is vital for confirming the safety and efficacy of the compound. The subchronic toxicity of gallic acid (GA) was assessed in F344 rats through a diet that included 0%, 0.2%, 0.6%, 1.7%, and 5% GA over a period of 13 weeks. In males, toxic effects were observed at doses of 0.6% or higher, while females exhibited effects at 5%. These effects included decreased hemoglobin concentration, hematocrit levels, and red blood cell counts, alongside an increase in reticulocyte counts. Histopathological examination revealed extramedullary hematopoiesis, hemosiderin deposition, and congestion in the spleens of animals treated with 5% GA, indicating the potential development of hemolytic anemia. Furthermore, both male and female rats receiving 1.7% GA exhibited centrilobular liver cell hypertrophy, which was also associated with increased liver weight. In the kidneys, Berlin blue-negative brown pigment deposition was noted in the proximal tubular epithelium of rats treated with 5% GA. However, the severity of these pathological changes was mild. According to the toxicology findings, a concentration of 0.2% was identified as the no-observed-adverse-effect level (NOAEL) in rats. This level was translated into 119 and 128 mg/kg/day, respectively, for male and female rats [231]. The gallic acid is non-toxic up to 5000 mg/kg body weight when given orally. Furthermore, the subacute study indicated the absence of cumulative toxicity, as reflected in the insignificant changes in the parameters examined. The NOAEL was 5000 mg/kg body weight, the highest dose examined [232]. Another study found that an 80% GA with some other herbal drugs, including rhubarb, astragalus, red sage, turmeric, and ginger, did not exhibit any reproductive toxicity in pregnant rats at dosages of 430 mg/kg/day or lower [233].

Despite the significant body of promising preclinical evidence for gallic acid, including its high safety margin demonstrated by a NOAEL of 5000 mg/kg in rats [232], translation into widespread clinical use remains limited. This gap can be attributed to several factors, including poor oral bioavailability, rapid metabolism and clearance, variability in effective dosing, physiological differences between experimental models and humans, limited human pharmacokinetic data, insufficient information on long-term safety, and long-term effects influencing the outcomes. The lacunae of large, well-designed clinical trials have further hindered the transition of gallic acid from experimental studies to clinical use. To move gallic acid forward as a therapeutic option, it will be indispensable to address these challenges through optimized formulations, enhanced delivery methods, and thorough clinical evaluations.

## 8. Clinical Study Based on Gallic Acid

A study was conducted to examine GA’s DNA protective potential in a placebo-controlled human intervention trial using single-cell gel electrophoresis. Supplementation of drinking water with GA (12.8 mg/person/d) for three days led to a noteworthy decrease of DNA migration attributable to oxidized pyrimidines as well as oxidized purines in lymphocytes of healthy individuals by 75% and 64%, correspondingly. Additionally, DNA damage induced by ROS treatment of the cells was reduced after consumption of GA (by 41%). These activities were paralleled by a decrease in intracellular ROS levels and in the activities of antioxidant enzymes in lymphocytes, whereas no alterations in the total antioxidant capacity, in malondialdehyde levels in serum, or in urinary excretion of isoprostanes were observed [234]. Another study was designed to find out if gallic acid (GA) prevents oxidative stress in diabetic patients. It was noticed that a noteworthy decrease in oxidized purines as well as pyrimidines was observed. Furthermore, the plasma concentrations of oxidized-LDL as well as C-reactive protein are reduced after the intervention by 24% and 39%, correspondingly. No changes of other biomarkers are noticed. A small amount of GA (in the range of daily consumption in Central Europe) prevents oxidative DNA damage. A modest intake of GA, similar to daily consumption levels in Central Europe, helps prevent oxidative DNA damage and as well as decreases markers, which reflect inflammation and increased risks of CVD and cancer [235].

## 9. Conclusions, Limitations, and Future Prospectives

Gallic acid is a polyphenolic compound commonly found in natural sources such as fruits and vegetables. It plays a significant role in managing various pathogenesis through different mechanisms. Gallic acid demonstrates antioxidant potential by scavenging reactive oxygen species and reducing malondialdehyde production. Additionally, its effectiveness in disease prevention and treatment has been supported by evidence demonstrating reduced levels of inflammatory mediators. Additionally, it is versatile in disease prevention, and treatment is supported by its role as hepatoprotective, neuroprotective, cardioprotective, anti-obesity, antimicrobial, and renoprotective. Additionally, gallic acid has demonstrated antidiabetic potential by enhancing insulin sensitivity, lowering glucose levels, improving redox status, reducing inflammation, and maintaining tissue architecture. This compound has also shown anticancer properties by modulating various cell signaling pathways. Synergistic effects of gallic acid in combination with other compounds or drugs have been reported, demonstrating enhanced pharmacological efficacy, including improved antioxidant, antimicrobial, and anticancer activities. Although gallic acid shows important promise as a therapeutic agent, its clinical use is hindered by numerous challenges. One major issue is its poor bioavailability as well as rapid metabolism, which meaningfully limit systemic exposure and, therefore, its effectiveness in vivo. Although many studies report promising results, most in vivo and in vitro experiments use relatively high doses, which may not be attainable or safe in humans. This raises important questions about dose translation, therapeutic window, and long-term safety. Additionally, the pharmacokinetic data for gallic acid are insufficient. There are few thorough studies examining its absorption, distribution, metabolism, and excretion, as well as limited information on its active metabolites. This inadequate data complicates the design of rational dosing regimens and the prediction of possible drug–drug interactions. Moreover, the mechanistic pathways of gallic acid are not fully understood in pathogenesis management. Although its antioxidant and anti-inflammatory effects are well-documented, the specific molecular targets, signaling pathways, and tissue-specific mechanisms are still unclear. It remains uncertain whether the observed benefits result from gallic acid itself or its metabolites, and how these mechanisms might vary across different diseases. Most of the existing evidence comes from preclinical models, highlighting the need for randomized controlled trials to properly assess efficacy, safety, and optimal dosing in humans. Lastly, the long-term toxicity and potential adverse effects associated with chronic administration of gallic acid have not been thoroughly investigated. Innovative approaches such as nanoparticles or targeted delivery systems may enhance bioavailability and tissue specificity, but their efficacy and safety must be validated. In summary, addressing these gaps through dose-scaling studies, advanced pharmacokinetic profiling, mechanistic research, optimized formulations, and extensive clinical trials will be important for translating gallic acid into a clinically viable therapeutic agent.

## Figures and Tables

**Table 1:** This table summarizes the hepatoprotective effect of GA by reduction of oxidative stress, inhibition of lipid peroxidation enzymes, and preservation of hepatocellular integrity.

**Hepatoprotective potential**	**Study Model**	**Doses**	**Findings of the Study**	**Refs.**
	Nitrosodiethylamine-induced liver inflammation model	100 mg/kg	° GA reversed increase in the total serum proteins and bilirubin toward normal levels and restores the level of antioxidant enzymes ° Liver tissue architecture was maintained, and collagen fiber was decreased	[70]
	Carbon-tetrachloride-induced hepatic damage model	50, 100 mg/kg	° GA decreases serum hepatic enzymes and up-regulate antioxidant gene expression ° Its inflammatory cytokines expression down-regulates	[49]
	Cyclophosphamide-induced hepatic damage model	100, 200, and 400 mg/kg	° GA protected the hepatic tissue ° It increased levels of liver GSH and SOD and maintained liver tissue architecture	[71]
	Mercuric-chloride-induced liver damage model	50, 200 mg/kg	° Gallic acid reduced the levels of liver function enzymes and lipid peroxidation ° Gallic acid treatment enhanced the activity of antioxidant enzymes	[72]
	Paracetamol-induced liver damage model	100 mg/kg	° Gallic acid reduced lipid peroxidation and inflammatory marker ° Increased the antioxidant enzymes	[73]
	Sodium-fluoride-induced liver injury model	10 and 20 mg/kg	° The pretreatment with gallic acid normalized the oxidative stress	[74]
	Carbon tetrachloride-induced liver damage model	50, 100 mg/kg	° The treatment with GA improved histopathologic changes ° The expression of p53 gene increased after GA treatments	[75]
	Diclofenac-induced liver toxicity model	50 and 100 mg/kg	° Total bilirubin and liver function enzymes levels decreased by GA ° Treatment with GA raised in antioxidant capacity and decreased MDA levels	[76]
	Sodium-fluoride-induced liver injury model	20 mg/kg	° Gallic acid protected liver damage by reduction in lipid peroxidation, increased antioxidant enzymes, and liver function enzymes	[77]
	CCl4-induced liver damage model	1 g/kg	° Gallic acid exhibited ability to protect mtDNA against depletion	[78]

**Table 2:** Neuroprotective potential of gallic acid through different mechanisms as reduction of oxidative stress, neuroinflammation, prevention of neural injury, and improving cognitive impairment.

**Neuroprotective effect**	**Study Type**	**Doses**	**Findings of the Study**	**Refs.**
	In vivo	100 mg/kg	° GA restored the total thiol and GPx contents and spatial memory ° GA showed activity against cognitive deficits	[79]
	In vivo	50, 100 mg/kg	° GA improved motor deficits and protect SNc neurons	[80]
		30 mg/kg	° GA inhibited cognitive deficits and biochemical alterations	[81]
	In vivo	30 mg/kg	° GA alleviated cognitive impairments of ° GA improved synaptic strength	[83]
	In vivo	100 mg/kg	° GA attenuated neurobehavioral disorders and neuronal loss	[43]
	In vivo	30 mg/kg	° Treatment with GA reversed the memory impairment ° Treatment decreased MDA level ° Increased the crossing and grooming activities ° Decreased the number of nuclear pyknosis	[84]
	In vivo	50 mg/kg	° GA decreased neuroinflammation ° GA reduced neuronal loss ° GA alleviated HIBD-induced cognitive impairment	[85]
	In vivo	40 mg/kg	° GA improved cognitive impairment and alleviate oxidative stress	[86]
	In vivo	20 mg/kg	° GA reversed the inhibition of brain activity ° GA reversed the brain levels of serotonin and dopamine	[87]

**Table 3:** Cardioprotective potential of gallic acid based on animal models through different mechanisms such as reduction of lipid profiles, prevention of myocardial injury, and enhancement of endothelial function.

**Cardioprotective activity**	**Study Model**	**Doses**	**Outcomes of the Study**	**Refs.**
	Isoproterenol-induced myocardial infarction model	15 mg/kg	° Cardiac marker enzymes, lipid peroxidation products decreased by GA ° Antioxidants enzymes increased by GA treatment	[88]
	Doxorubicin-induced myocardial toxicity model	15, 30 mg/kg	° GA restored the endogenous antioxidant system	[89]
	Isoproterenol-induced cardiotoxicity model	50 mg/kg	° GA increased cardiac function. ° It suppressed LDH and CK-MB levels and cardiac hypertrophy	[90]
	Cardiomyocytes	10 µm	° GA blocked cardiomyocyte dypertrophy	[91]
	Cardiac hypertrophy and dysfunction model	5 or 20 mg/kg	° GA ameliorated myocardial fibrosis, oxidative stress and inflammation	[91]
	Doxorubicin-induced cardiotoxicity model	200 mg/kg	° Antioxidant enzyme activities increased by GA ° TNF-α and Cox-2 expression reduced, pathologic tissue damage reduced by GA	[92]
	Isoproterenol-induced myocardial infarcted model	15 mg/kg	° Pretreatment of maslinic acid and GA reversed XO enzyme, indicating protective action against myocardial necrosis	[93]
	Cadmium-induced cardiac remodeling model	15 mg/kg	° GA attenuated lipid peroxidation and cardiac antioxidant enzymes enhanced ° Fibrotic proliferation and ventricular hypertrophy decreased	[94]
	Isoproterenol-induced cardiotoxicity model	15 mg/kg	° GA prevented changes in lipid peroxidation products, activities of cardiac marker enzymes	[95]
	Cardiac ischemia/reperfusion injury model	30 mg/kg	° Heart damage markers, including infarct size, ROS, and SGK1 gene expression reduced by GA	[96]
	DOX-induced cardiac toxicity model	60, 120 mg/kg	° GA pretreatment alleviated ECG abnormalities	[97]

**Table 5:** Antidiabetic potential of gallic acid based on animal model and through different mechanisms. This table summarizes the effects of GA in the management of diabetes by improvement of insulin sensitivity, enhancement of glucose uptake, and protection of pancreatic β-cells.

	Study Model	Doses	Finding of the Study	Refs.
**Antidiabetic activity**	Diabetic nephropathy model	30 mg/kg	° GA improved diabetic nephropathy via amelioration of biochemical indices, oxidative stress ° It showed its role in the amelioration of histopathological aspects and fibrosis	[112]
	Diabetic model	100 mg/kg	° GA improved hyperinsulinemia and hepatic steatosis ° GA ameliorated hepatic lipid peroxidation and antioxidant enzymes increased	[113]
	Streptozotocin-induced diabetes model	20 mg/kg	° The pancreas, liver, and kidney tissue degradation rescued by GA and AGP treatment. ° Administration of GA restored GLUT4 protein expression	[114]
	Streptozotocin-induced diabetic model	25 mg/kg	° GA improved the free radical scavenging property	[116]
	Diabetic kidney disease model	30 mg/kg	° The combination of GA and MET showed renal protective effect	[117]
	Streptozotocin-induced diabetic model	10, 20mg/kg	° GA decreased pathological changes of pancreas	[118]

**Table 7:** The role of gallic acid in different pathogenesis through different mechanisms. This table summarizes the therapeutic role of GA in different diseases.

Activity	Study Types	Animal/Cell Lines	Dose	Outcomes	Refs.
Anti-obesity	In vivo	Mice	15, 45 mg/kg	° GA suppressed the elevation of blood triglyceride ° The BTE and GA groups exhibited suppression of weight gain	[145]
Anti-arthritis	In vivo	Rabbit	80 μM	° GA ameliorated cartilage damage in osteoarthritis model	[148]
Anti-colitis	In vivo	Mice	40, 80, 120 mg/kg	° Crypt architecture and colon damage was restored by GA	[151]
	In vivo	Mice	10 mg/kg	° GA attenuated colitis and reduced histological changes	[152]
	In vivo	Mice	10 mg/kg	° GA attenuated the colon shortening ° GA reduced the histopathological changes	[153]
Role in pulmonary inflammation and emphysema	In vivo	Mice	200 mg/kg	° GA suppressed the neutrophil infiltration and restored the redox imbalance	[156]
Anti-asthma	In vivo	Mice	100 mg/kg	° The pro-inflammatory cell infiltration was reduced and airway hyperresponsiveness improved by GA	[58]
Protective role in lung	In vivo	Rats	50, 100, 200 mg/kg	° GA attenuated oxidative damage and fibrosis	[157]
Role in polycystic ovary syndrome	In vivo	Mice	75 mg/kg	° GA may serve as a probable therapy for managing PCOS by regulating endocrine and metabolic disturbances.	[158]
Protective effects on testicular and epididymal damage	In vivo	Rats	50 mg/kg	° GA improved reproductive toxicity and restored structural as well as functional deterioration	[159]
Role in wound healing	In vivo	Rats	1.2 mg/mL	° GA treatment improved healing	[160]
Antihypertensive	In vivo	Mice	5 or 20 mg/kg	° GA decreased ang II-Induced hypertension and inflammation	[161]
Role in radioprotection	In vitro	Lymphocytes	25 g/mL	° Gallic acid protected lymphocytes from gamma radiation-induced DNA destruction	[162]
	In vivo	Mice	100 mg/kg	° GA inhibited chromosomal aberrations and micronucleus formation	[163]
Role in cornea protection	In vivo	Rats	10 mg/kg	° Supplementation with gallic decreased inflammation, and alteration of their cornea histology	[164]

**Table 8:** The effect of gallic acid on inhibiting microorganisms. The table describe the inhibitory effects of GA on microorganisms through mechanisms of action such as membrane disruption, inhibition of biofilm formation, and interference with metabolic pathways.

Activity	Key Findings	Refs.
Antibacterial	° GA exhibited antibacterial activity against bacterial strains ° It effectively diminished bacterial growth and metabolic activity	[180]
	° GA revealed bactericidal potential ° It inhibited the formation of bacterial biofilm ° GA caused damage to both outer and inner membranes	[181]
	° GA possesses antimicrobial properties and also enhances the effectiveness of antibacterial agents	[182]
	° GA exhibited stronger antibacterial activity against bacteria	[183]
	° GA directed to inhibitory potential, which was revealed by reduced cell viability and damaged cell membranes ° GA inhibited biofilm formation	[184]
	° GA inhibited biofilm formation	[185]
Antiviral	° GA reduced the replication of HSV-2 ° Anti-HSV-2 activity of GA was recognized for its virucidal potential on virus particles	[186]
	° GA suppresses H1N1 influenza viral infectivity via autophagy pathway restoration	[187]
	° GA downregulated the expression levels of HCV-RNA as well as NS5A-HCV protein	[188]
	° GA was a powerful inhibitor of influenza virus mRNA replication ° GA inhibited neuraminidase activities and viral glycoprotein	[189]
	° GA inhibited the proliferation of HPVep cells	[190]
	° GA exhibited a strong antiviral effect against various viruses	[191]
Antifungal	° GA was effective in inhibiting the growth of various C. auris strains	[192]
	° GA showed a wide spectrum of antifungal activity	[193]
	° GA caused alterations of the mitochondrial transmembrane potential, membrane integrity, externalization of phosphatidylserine	[194]
	° GA showed high antifungal activity against F. solani	[195]
	° GA significantly inhibited the growth of A. fumigatus, as well as its biofilm formation and adhesion	[196]

**Table 9:** Synergistic effects of gallic acid with other compound/drugs. The table summarizes the synergistic interactions of GA with other compounds/drugs. It highlights the combined effects on enhancing pharmacological efficacy, improving antimicrobial and anticancer activities.

	Compound/Drug	Study Type	Activity	Outcomes	Refs.
**Gallic acid**	Curcumin	In vitro	Anticancer	° The combination reduced the growth of breast cancer cells ° It enhanced the sub-G1 cell population and apoptotic cells were increased	[197]
	Curcumin	In vitro	Antioxidant	° Curcumin demonstrated a synergistic antioxidant effect when used in conjunction with gallic acid	[198]
	Hesperidin	In vitro	Anticancer	° The combined treatment showed a greater inhibitory effect on cancer cell viability	[200]
	Azithromycin	In vitro	Antibacterial	° The combination of Azithromycin/gallic acid showed additive effects against P. aeruginosa as well as MSSA ° Synergistic effect against MRSA	[201]
	Paclitaxel and carboplatin	In vitro	Anticancer	° The triplet combination significantly increased the mRNA expression of Bax, P53, and CASP-3	[202]
	Temozolomide	In vitro	Anticancer	° TMZ/GA combination increased the inhibition of cellular viability as well as apoptotic level	[203]
	Cisplatin	In vitro	Anticancer	° GA in the combination with cisplatin caused synergistic potential on pro-apoptotic mediators	[204]
	Cisplatin	In vitro	Anticancer	° GA synergistically reduced cancer cell viability compared to the individual therapies ° The frequency of micronuclei decreased in combinational therapy	[125]
	Cisplatin	In vitro	Anticancer	° GA increased the anticancer role of cisplatin ° It also caused cell apoptosis induction	[205]
	Pirarubicin	In vitro	Anticancer	° Gallic acid (GA)/Pirarubicin (Pira) combination reduced cell viability and mitochondrial activity	[206]
	Famotidine	In vivo	Antiulcer	° The combinations showed a role in the protection of the gastric mucosa ° The combination treatment increased levels of antioxidant enzymes, decreased myeloperoxidase and lipid peroxidation	[207]

## Data Availability

No new data were created or analyzed in this study. Data sharing is not applicable to this article.

## Abbreviations

AGEs	Advanced Glycation End Products
ALP	Alkaline Phosphatase
ALT	Alanine Transaminase
ASCVD	Atherosclerotic Cardiovascular Disease
AST	Aspartate Aminotransferase
CCL4	Carbon Tetrachloride
CDDP	Cisplatin
COX-2	Cyclooxygenase-2
EPM	Elevated Plus-Maze
GA	Gallic Acid
GPx	Glutathione Peroxidase
GR	Glutathione Reductase
GSH	Glutathione
HFD	High-Fat Diet
HO-1	Heme Oxygenase-1
IL-1β	Interleukin-1 Beta
IL-6	Interleukin-6
ISO	Isoproterenol
MBC	Minimum Bactericidal Concentration
MDA	Malondialdehyde
MFST	Modified Forced Swim Test
MIC	Minimum Inhibitory Concentration
MMP	Mitochondrial Membrane Potential
MMPs	Matrix Metalloproteinases
MTX	Methotrexate
NF-κB	Nuclear Factor Kappa-B
NRF2	Nuclear Factor Erythroid 2–Related Factor 2
NSCLC	Non-Small Cell Lung Carcinoma
OVA	Ovalbumin
PLGA	Poly(D,L-lactide-co-glycolide)
PPE	Porcine Pancreatic Elastase
ROS	Reactive Oxygen Species
SOD	Superoxide Dismutase
STZ	Streptozotocin
TMZ	Temozolomide
TNF-α	Tumor Necrosis Factor-Alpha
VEGF	Vascular Endothelial Growth Factor
WHO	World Health Organization
XO	Xanthine Oxidase
